# Cholesteryl esters and high protein-to-lipid ratios distinguish non-vesicular extracellular particles from extracellular vesicles

**DOI:** 10.1186/s12964-026-02880-6

**Published:** 2026-04-17

**Authors:** Sayam Ghosal, Rita Leporati, Árpád Varga, Petra Susanszki, Nóra Fekete, Tamás László, Tünde Bárkai, Fülöp Károly Grébecz, Delaram Khamari, Tárek Zoltán Magyar, Marcus Hoering, Krisztina V Vukman, Bernadett R Bodnár, Brachyahu M Kestecher, Mohamed A Fattah, Csaba Bödör, József Maléth, Gerhard Liebisch, Evelyn Orsó, Edit I Buzas, Xabier Osteikoetxea

**Affiliations:** 1 HCEMM-SU Extracellular Vesicle Research Group, Budapest, 1089 Hungary; 2 Institute of Genetics, Cell- and Immunobiology, Semmelweis University, Budapest, 1089 Hungary; 3 Division of Medical Oncology, Modena University Hospital, Modena, 41124 Italy; 4 First Department of Internal Medicine, University of Szeged, Szeged, 6725 Hungary; 5 Institute of Behavioural Sciences, Semmelweis University, Budapest, 1089 Hungary; 6 Department of Pathology and Experimental Cancer Research, Semmelweis University, Budapest, 1085 Hungary; 7 Institute of Clinical Chemistry and Laboratory Medicine, Regensburg University Hospital, Regensburg, 93053 Germany; 8 HUN-REN-SU Translational Extracellular Vesicle Research Group, Budapest, 1089 Hungary

**Keywords:** Extracellular vesicle, non-vesiclular extracellular particle, exomere, cholesteryl ester, dSTORM

## Abstract

**Background:**

Mechanisms governing non-vesicular extracellular particle (NVEP) biogenesis and compositional diversity remain incompletely characterized.

**Method:**

This study integrates multiparametric profiling to define NVEPs. NVEPs were isolated by differential ultracentrifugation and characterized using complementary biochemical, lipidomic, and imaging approaches, including protein-to-lipid ratio analysis, lipid profiling, and advanced microscopy.

**Result:**

Comprehensive characterization demonstrated that NVEPs exhibit significantly elevated protein-to-lipid ratios compared to extracellular vesicles (EVs) subtypes. Lipidomic profiling revealed pronounced enrichment in cholesteryl esters and triacylglycerols, with distinct carbon chain length signatures differentiating NVEPs from small EVs. Super-resolution microscopy confirmed marker heterogeneity across populations, with NVEPs showing specific enrichment of Arf6 and CD63.

**Conclusion:**

These findings provide a robust compositional framework to distinguish non-vesicular extracellular particles from canonical EVs and underscore their emerging significance as mediators of intercellular communication and potential vehicles for targeted delivery.

**Supplementary Information:**

The online version contains supplementary material available at 10.1186/s12964-026-02880-6.

## Introduction

The field of extracellular vesicle (EVs) research has progressed significantly in recent decades, elucidating the complex nature and increasing potential of EVs for disease diagnosis and therapy [1]. EVs have demonstrated dual functions, influencing both the progression and inhibition of various conditions including cancer [2], cardiovascular disorders [3, 4], and neurological diseases [5]. Current understanding categorizes EV biogenesis broadly into two primary pathways: direct budding from the plasma membrane (ectocytosis) or the formation of intraluminal vesicles (ILVs) within multivesicular bodies (MVBs), which are endosomal compartments that contain ILVs formed by inward budding of the limiting membrane and subsequently released into the extracellular space as exosomes (exocytosis) [6]. This diverse origin results in a heterogeneous population of EVs, occasionally with relatively defined small or large sizes, leading to substantial debate regarding their precise sources. Small EVs (~ 50–200 nm diameter, sEVs) are primarily secreted through the exosomal pathway, while they postulate that large EVs (> 200 nm diameter, lEVs) are predominantly released by ectocytosis [7]. The discovery of non-vesicular extracellular particles (NVEPs), including exomeres [8] and supermeres [9], recently introduced further complexity in biogenesis, whose biogenesis mechanisms remain unknown. NVEPs merit broad scientific focus because they are highly abundant, carry disease-linked proteins and RNAs once ascribed to EVs, and play key roles in intercellular signaling and clinical applications [10, 11]. Accurate profiling NVEP is essential to decipher cellular secretory pathways and to tailor biomarker discovery and therapeutic delivery, marking this effort as a pivotal frontier in cell biology and translational medicine.

Questions persist as to whether NVEPs follow conventional EV release pathways or arise through distinct biogenetic mechanisms. Their molecular composition and origin remain poorly defined, largely due to the absence of systematic, multiparametric comparisons with canonical EV subtypes. Given these unresolved issues, continued investigation into the origin, structure, and function of both EVs and NVEPs is essential to refine current classification frameworks and fully understand their biological roles.

In this study, we sought to clarify the molecular distinction and potential biogenetic relationships among lEVs, sEVs, and NVEPs by focusing on three extracellular fractions that have been consistently described across prior differential centrifugation and filtration based studies [9, 12–14]. To enable meaningful comparison of NVEPs with canonical EV subtypes, we grounded our investigation in an orthogonal multiparametric framework informed by published analyses of particle morphology, molecular composition, and single-particle behavior. To further interrogate the putative origins of these populations, we drew upon a panel of membrane-trafficking markers previously implicated in distinct secretory routes—including membrane-associated motifs such as Myristoylation/Palmitoylation (MysP) [15, 16], the canonical exosomal marker CD63 [17–19] and regulators associated with ectosomal shedding or endolysosomal trafficking such as Arf6 [20], non-canonical EV markers like Rab GTPase 7a (Rab7a) [21, 22], and microtubule associated protein 1 light chain 3 beta (LC3b) [23–26], which recent studies demonstrate are involved in alternative EV release pathways. Motivated by recent evidence suggesting that NVEPs may participate in intercellular communication, we also considered the conceptual basis for their potential involvement in molecular cargo delivery, which remains an important open question in the field.

## Materials and methods

### Cell culture

In this study, two derivatives of human embryonic kidney cells (HEK293) were utilized: HEK293T (passage number 12–30) and 293 A (passage number 5–12) cell lines. HEK293T cells were obtained from the European Collection of Authenticated Cell Cultures (ECACC). 293 A cell line was provided by Várnai Péter (Semmelweis University, Budapest, Hungary). All cells were cultured in Dulbecco’s Modified Eagle’s Medium (Catalogue # D6046; Merck Group, MA, USA) supplemented with 10% fetal bovine serum (Catalogue # 10270106; Gibco, NY, USA) and 1% penicillin–streptomycin (Catalogue # P4333; Merck Group, MA, USA). All cells were incubated at 37 °C in a 5% CO_2_ environment and ≥ 90% humidity. Cells were passaged three times per week. Polymerase chain reaction (PCR) was routinely employed to assess cell cultures for mycoplasma contamination. The PCR primers utilized in this process were derived from previous studies [27] and have the following sequence: GTTTGATCCTGGCTCAGGAYDAAC and GAAAGGAGGTRWTCCAYCCSCAC.

### Molecular cloning

In this study, we have used 2 recombinant plasmids, which were developed in-house. They are Myc_EGFP_Arf6_HA (SGb42) (Fig. S1 A) and MysP-EGFP (BMKb9) (Fig. S1 B). We have also used pEGFP-LC3 (addgene: Plasmid #24920, MA, USA), EGFP-rab7 WT (addgene: Plasmid #12605, MA, USA), and CD63-pEGFP C2 (addgene: Plasmid #62964, MA, USA). Assembly cloning was performed using pcDNA3-EGFP (addgene: Plasmid #13031, MA, USA) vector inserting BRET from pRetroX-Tight-MCS_PGK-GpNLuc (addgene: Plasmid #70185, MA, USA). Vector was amplified with the primer shown (Supplementary Table T1. I. a, b of vector) and insert was amplified with primers shown (Supplementary Table T1. I. a, b inserts) resulting in pcDNA-BRET. The plasmid pcDNA-BRET was amplified with the primers shown (Supplementary Table T1. I. c, d of vector) and insert CyPET-Arf6 (addgene: Plasmid #18840, MA, USA) was amplified with the primers (Supplementary Table T1. (I) c, d of insert) resulting in SG2. SDM method was exploited to design BMKb9 (MysP-EGFP) by adding the MysP construct upstream of the EGFP cassette in the pcDNA3.1 + vector (addgene: Plasmid #13031, MA, USA) using forward and reverse primer shown (Supplementary Table T1. (II) a). The molecular cloning was validated through Sanger sequencing. The single amino acid sequence of the recombinant plasmid are shown in Supplementary Table T2. Upon publication the plasmids will be made available to other researchers through Addgene and can also be directly shared by the authors upon request.

### Cell transfection

Transfections were conducted utilizing the chemical transfection reagent Polyethylenimine (PEI) “Max” (catalogue #24765-1, Polysciences, Inc., PA, USA) prepared at a concentration of 1 mg/mL. A 1:3 ratio of DNA to PEI was maintained throughout the transfection procedure. Cells were seeded to achieve approximately 30% confluency prior to transfection. The transfection mixture was prepared in Opti-MEM™ (catalogue # 31985062, Gibco, NY, USA) and incubated at 37 °C for 30 min before being added dropwise to the cells, followed by overnight incubation. The media was subsequently replaced with complete media to minimize cellular toxicity. Transfection efficiency was evaluated for 36 h post-transfection using a CytoFLEX S N2-V3-B5-R3 Flow Cytometer (catalogue #B78557, Beckman Coulter, Inc, IN, USA) and CytExpert software (version 2.4.0.28, Beckman Coulter, Inc, IN, USA). To control for variations in cell number and viability post-transfection, EV particle concentrations measured by NTA were normalized to the count of viable cells at harvest. Cell viability was confirmed using Trypan Blue exclusion; only experiments maintaining > 90% viability were included. Additionally, transfection efficiency was evaluated by flow cytometry, and samples exhibiting < 75% efficiency based on fluorescence signal were excluded from downstream analysis to ensure experimental consistency.

### Confocal microscopy and membrane association measurement

Before analysis by confocal microscopy, the 293 A cells (passage number between 5 and 12) were cultured on the surface of gelatin-fibronectin coated glass coverslips (VWR, International, PA, USA) in 24 well plates (seeding density of 3.5E + 04 cells per well). The coating solution contained 0.1% gelatin (Merck Group, MA, USA) and 1 mg/mL fibronectin (Invitrogen, MA, USA). After overnight incubation with coating solution at 37 °C, it was washed once with DMEM and 293 A cells were seeded to achieve 30% confluency during the transfection. Following 36 h of incubation, cells were fixed with 4% paraformaldehyde (PFA) (VWR, International, PA, USA) in 1X PBS for 20 min at room temperature, followed by extensive washing in 1X PBS. Samples were mounted in ProLong Diamond with 4’,6-diamidino-2-phenylindole (DAPI) (Invitrogen, MA, USA) and examined using a Leica SP8 Lightning confocal microscope. Image analysis and processing was performed using Leica LASX software. Several images were obtained from 5 different cell passages, which considered as 5 biological repeats that was used for downstream image analysis to determine the membrane association percentage.

For membrane association analysis, we followed pipeline, which begins from the output of the confocal images in .tif format. The pipeline is explained well as flowchart shown in Fig. S2. In brief, we first ensure the dimension of the image to be processed downstream in 1442 × 1442 pixel using ImageJ 1.54 g. Later, manually we outline the plasma membrane from the brightfield image and the nuclear membrane from the blue fluorescence image using Adobe Fresco version 7.0.1. The membrane contour is created by running python script in Visual Studio Code Version: 1.106.2 (Universal). Later all the images including for single confocal experiment are separately saved in four different folders: Blue, Green, Merged, Contours. These folders are run in the Cell Profiler platform (version 4.2.8). The output obtained was run in the python script for decoding the membrane association of the marker and the generate the debugged segmented image and showing the positive marker excluding any artificial marker. Two python scripts and Cell Profiler work file can be shared upon genuine request.

We determined the membrane association for each marker by comparing the relative distance to nucleus or membrane, presenting representative images of fluorescence (FL), brightfield (BF), and segmented confocal micrographs of individual cells (Fig. 4A). For this analysis, we utilized the mean of four optimal confocal micrographs exhibiting normal cell morphology. The formula employed to determine the Membrane association percentage was as follows:

$$\mathrm{Membrane}\;\mathrm{association}\;\%=\left\{\frac{\mathrm{Dn}}{\mathrm{Dn}\;+\;\mathrm{Dm}}\right\}\times100\%$$

The mean Membrane association % of mean 4 confocal micrographs (which is of 4 biological repeats) of all markers were plotted using Prism 10 for macOS version 10.3.1 (464). Native cells without any transfection were used as negative controls, showing no EGFP labelled marker to compare the subcellular localization and membrane-association measurements of EGFP labelled markers.

### Immunofluorescence analysis of endogenous compartment markers

To evaluate whether EGFP overexpression influenced protein localization, we performed immunofluorescence staining of endogenous compartment markers (WT) under identical experimental conditions. Cells were fixed by removing the culture medium, rinsing once with PBS, and incubating with 4% paraformaldehyde (VWR, International, PA, USA) for 20 min at room temperature. After fixation, we washed cells three times with 1X PBS. We permeabilized cells with 0.1% Triton X-100 (T9284-500ML; Merck Group, MA, USA) in PBS for 5 min at room temperature and washed them three times with PBS. We then blocked nonspecific binding sites by incubating samples in 1% bovine serum albumin (BSA) in PBS for 30 min at room temperature on rocker. Primary antibodies (Table 1) were prepared as specified and applied in antibody dilution buffer (0.1% BSA in PBS). Samples were incubated with the primary antibodies overnight at 4 °C. After incubation, cells were washed three times with PBS and blocked again with 1% BSA for 15 min at room temperature, followed by three PBS washes. Secondary antibodies (Table 1) were subsequently applied as specified and incubated for 1 h at room temperature on a rocker. Following incubation, cells were washed twice with PBS and once with double-distilled water. Coverslips were mounted on glass slides using ProLong Diamond with 4’,6-diamidino-2-phenylindole (DAPI) (Invitrogen, MA, USA) to counterstain nuclei. We acquired images with a Leica SP8 Lightning confocal microscope and performed image acquisition and processing using Leica LAS X software.

**Table 1:** Primary and secondary antibodies and working conditions for confocal microscopy, immuno-gold TEM, and dSTORM analyses

Target protein / Antibody	Host	Supplier	Catalog No.	Dilution	Working concentration
Primary antibodies					
Arf6	Mouse	Thermo Fisher Scientific	MA3-060	1:100	ND
CD63– Alexa Fluor™ 647	Mouse	Unchained Labs	251–1044	1:200	ND
CD9– Alexa Fluor™ 488	Mouse	Unchained Labs	251–1044	1:200	ND
CD81– Alexa Fluor™ 555	Mouse	Unchained Labs	251–1044	1:200	ND
CD63 (0.5 mg/mL)	Mouse	Thermo Fisher Scientific	10628D	1:200	2.5 µg/mL
LC3B (1 mg/mL)	Mouse	Thermo Fisher Scientific	MA5-31541	1:200	5 µg/mL
Rab7a	Rabbit	Merck Group	ZRB1120	1:1000	ND
ApoB	Synthetic	Tresars	TR86843-02	1:200	ND
Hsp90β (0.5 mg/mL)	Mouse	Thermo Fisher Scientific	379,400	1:200	2.5 µg/mL
anti-GFP (2 mg/mL)	Rabbit	Thermo Fisher Scientific	A11122	1:200	10 µg/mL
Mouse IgG isotype control (0.05 mg/mL)	Mouse	Abcam	ab91356	1:10	5 µg/mL
Secondary antibodies					
Goat anti-Mouse IgG (H + L), Alexa Fluor™ 568 (2 mg/mL)	Goat	Thermo Fisher Scientific	A11004	1:400	5 µg/mL
Goat anti-Rabbit IgG, Alexa Fluor™ 568 (2 mg/mL)	Goat	Thermo Fisher Scientific	A-11,011	1:400	5 µg/mL
Goat anti-Mouse IgG, Alexa Fluor™ 488 (2 mg/mL)	Goat	Thermo Fisher Scientific	A-11,001	1:400	5 µg/mL
Donkey anti-Rabbit IgG (H + L), Alexa Fluor™ 647 (2 mg/mL)	Donkey	Thermo Fisher Scientific	A-31,573	1:400	5 µg/mL

*ND*, concentration information unavailable from supplier datasheet

### Isolation of EVs and NVEPs

HEK293 cells (5 × 10⁶ cells per 150 mm cell culture plates; passage numbers 12–23) were seeded in 150-mm dishes containing 20 mL serum-free medium and incubated for 24 h. EVs and NVEPs were then isolated with modifications to a previously described method [9, 28]. This process was done for 4 different passages which were considered as 4 biological repeats. Briefly, cells were removed by centrifugation at 500 ×g for 10 min at room temperature, followed by gravity filtration of the supernatant through a 5 μm filter (Merck Group, MA, USA), which was centrifuged (Hermle Z326 K Refrigerated Centrifuge, Hermle AG, Gosheim, Germany) at 2,000 ×g for 20 min at 4 °C. The supernatant obtained was filtered using 0.8 μm filter (Merck Group, MA, USA) and was centrifuged with a Hermle Z326 K Refrigerated Centrifuge (Hermle AG, Gosheim, Germany) equipped with fixed angle rotor 220.78 (gmax = 20,984; k factor = 597, Hermle AG, Gosheim, Germany) at 14000 xg for 40 min at 4 °C.The pellet obtained was washed with the same centrifugation parameter resulting in 14k-lEV. The resulting supernatant was filtered through a 0.2 μm filter (Merck Group, MA, USA) and subsequently ultracentrifuged at 100,000 × g for 2.5 h at 4 °C using an Optima XPN-100 Ultracentrifuge (Beckman Coulter, Inc., CA, USA) equipped with a SW32Ti rotor (gmax = 98,058; k factor = 343, Beckman Coulter, Inc., CA, USA). The pellet obtained was washed with the same centrifugation parameter resulting in 100k-sEV and its supernatant was ultracentrifuged at 167,000 × g for 16 h at 4 °C using an Optima XPN-100 Ultracentrifuge (Beckman Coulter, Inc., CA, USA) equipped with a SW32Ti rotor (gmax = 166,782; k factor = 215, Beckman Coulter, Inc., CA, USA) and pellet was washed using same centrifugation parameter resulting in 167k-NVEP.

### TEM and Immuno-EM of EVs and NVEPs

For TEM imaging, we applied the previously described EV isolation method with minor modifications [16]. To isolate the EV subtypes and 167k-NVEPs, nine 150 mm cell culture plates (seeding density of 5E + 06 cells) with single passage number in serum depleted media followed by isolation of EV subtypes and 167k-NVEPs as mentioned above in method Sect. 5 and it was done for 3 biological repeats. Previously centrifugated concentrated 14k-lEVs, 100k-sEV and 167k-NVEPs pellets were resuspended in 0.1 filtrated 10 µL 1× PBS instead of 50 µL final volume. Samples were fixed in 2% paraformaldehyde (Paraformaldehyde, powder, 95%, 158127, Sigma Aldrich, St. Louis, MO, USA) in 1× PBS for 30 min at 4 °C. Then, they were incubated for 15 min on glow-discharged at 7.2 V for 60 s, carbon-coated 100-mesh copper grids using a Bal-Tec MED 020 Coating System. After washing with 1× PBS and fixating with glutaraldehyde 1% (Sigma-Aldrich, St. Louis, MO, USA) for 5 min at 4 °C, grids were washed again and dried with filter paper. The grids were treated with 2% aqueous uranyl acetate (Sigma-Aldrich, St. Louis, MO, USA) for 2 min to generate negative staining. Samples were washed and dried, then analyzed with FEI Tecnai G2 Spirit TEM (Thermo Fisher Scientific, MA, USA) equipped with a Morada digital camera (Olympus Soft Image Solutions GmbH, Muenster, Germany).

For immuno-gold TEM, 8 µL of samples were incubated in 2% paraformaldehyde-PBS for 30 min and placed on grids for 15 min. Grids were washed with PBS, blocked with 0.1 M glycine and 0.3% BSA, and incubated with anti-CD63 primary antibody (Table 1) for 1 h, followed by secondary antibodies conjugated to 6 nm gold particles. After washing, a standard negative staining procedure was performed. Grids were imaged using a FEI Tecnai G2 Spirit TEM with a Morada digital camera. All TEM procedures were undertaken at the University of Valencia’s Electron Microscopy facility.

### Size, concentration, and zeta potential determination of EVs

Nanoparticle tracking analysis (NTA) was performed for 14k‑lEV and 100k‑sEV samples for 4 biological repeats (seeding density of 5E + 06 cells per 150 mm cell culture plates). NTA was not used for quantitative characterization of the 167k-NVEP fraction because these particles were close to or below the reliable size and light-scattering detection limits of our NTA setup, which was optimized and calibrated for conventional small and large EV subtypes (Fig. S4 A). Particles with diameters below approximately 60 nm are generally not reliably detected by NTA, and the practical lower detection limit for low–refractive-index lipid vesicles has been reported to lie in the ~ 70–90 nm range [29, 30]. Consistent with these limitations, NTA profiles obtained for the 167k-NVEP fraction were indistinguishable from the background signal found in PBS alone controls under our measurement conditions and did not yield robust quantitative data. Size distributions and concentrations of 14k-lEV and 100k-sEV were assessed using nanoparticle tracking analysis (NTA) on a ZetaView PMX120 instrument (Particle Metrix, Germany). Samples were diluted between 1000-fold to 20000-fold in 1X PBS to achieve optimal concentration (50–200 particles/frame) and analyzed across 11 positions with each position was recorded using two sequential measurement cycles for averaging. The following camera settings were used: shutter speed of 100, sensitivity of 70 (for 14k-lEV) and 80 (for 100k-sEV), and a frame rate of 30 frames per second.

The Zeta potential, a measure of the electrokinetic surface charge of nanoscale particles in suspension, reflects the stability of colloidal systems and provides insight into the physicochemical properties governing EV interactions with their surrounding environment. The zeta potential of 14k-lEV and 100k-sEV was measured three biological repeats (seeding density of 5E + 06 cells per 150 mm cell culture plates) at 25 °C with the following settings: sensitivity of 85, shutter value of 70, and a frame rate of 30 frames per second. ZetaView software (S/N: 19–459 ZetaView Particle Tracking Analyzer PMX-120 instrument, Munich, Germany, employing the ZetaView Analysis 8.05.12 SP2 software) was employed for data collection and analysis.

### Bead-based multiplex flow cytometry assay

Bead-based multiplex analysis was conducted using the MACSPlex EV Kit IO human (catalogue # 130-122-209, Miltenyi Biotec, Germany). Assays were performed as described earlier [31, 32]. EV and NVEP samples (isolated from Hek293 cells growing with seeding density of 5E + 06 cells per 150 mm cell culture plates) having 4 µg of total protein content measured by micro-BCA kit (catalogue # A55864, Thermo Fisher Scientific, MA, USA), diluted with MACSPlex buffer to a total volume of 120 µl and incubated with 15 µl of MACSPlex exosome capture beads overnight in wells of a pre-wet and drained MACSPlex 96-well 0.22 μm filter plate on an orbital shaker at 450 rpm at room temperature and washed with 200 µl MACSPlex buffer. For counterstaining of captured EVs and NVEPs, a mixture of APC-conjugated anti-CD9, anti-CD63 and anti-CD81 detection antibodies (supplied in the MACSPlex kit, 5 µl each) was added to each well in a total volume of 135 µl, and the plates were incubated on an orbital shaker at 450 rpm for 1 h at room temperature. Next, the samples were washed twice, resuspended in MACSPlex buffer and analysed by flow cytometry with a CytoFLEX S N2-V3-B5-R3 Flow Cytometer (catalogue # B78557, Beckman Coulter, Inc, IN, USA) and CytExpert software (version 2.4.0.28, Beckman Coulter, Inc, IN, USA).

### Single-particle interferometric reflectance imaging sensor (SP-IRIS)

The Leprechaun Exosome Human Tetraspanin capture Kit (catalogue # 251–1044, Unchained Labs, CA, USA) was utilized to capture 100k-sEVs and 167k-NVEPs (isolated from Hek293 cells growing with seeding density of 5E + 06 cells per 150 mm cell culture plate in serum depleted media) positive for CD81, CD9, and CD63, following previously established protocols [33]. In brief, approximately 1E + 06 particles in 50 µL were applied to background-scanned chips and left to incubate for 1 h at room temperature. The Leprechaun instrument scanned the chips using both interferometric microscopy and fluorescence channels corresponding to CD81, CD9, and CD63 signals. Leprechaun Analysis v2.1.1. software was employed to analyze the acquired images for particle concentration and size. 100k-sEV and 167k-NVEP were categorized into seven subpopulations based on their tetraspanin expression: CD9+, CD81+, CD63+, CD9 + CD81+, CD9 + CD63+, CD63 + CD81+, CD9 + CD81+CD63+.

To evaluate the potential presence of lipoprotein-associated particles within the 167k-NVEP fraction, we performed an additional SP-IRIS analysis using ApoB labeling. Briefly, 167k-NVEPs isolated from HEK293 cells cultured in serum-depleted medium were captured on Leprechaun chips functionalized with anti-CD63 and anti-CD81 antibodies provided in the Human Tetraspanin Capture Kit. Instead of CD9 detection, we incubated samples with ApoB-FITC antibody (Tresars, catalogue # TR86843-02, UK) to identify ApoB-associated particles (Tabe T1). Approximately 1E + 06 particles in 50 µL were applied to background-scanned chips and incubated for 1 h at room temperature. Chips were scanned using interferometric imaging together with fluorescence detection channels corresponding to CD63, CD81, and ApoB signals. Leprechaun Analysis v2.1.1 software was used to determine particle size distribution, concentration, and co-localization frequencies between tetraspanin-positive and ApoB-positive particles.

### Protein and lipid content determination

The total protein concentration of EV and NVEP preparations was quantified using the Micro BCA Protein Assay kit (catalogue # A55864, Thermo Fisher Scientific, MA, USA) according to the manufacturer’s instructions as previously described [28]. EV and NVEP samples (isolated from Hek293 cells growing with seeding density of 5E + 06 cells per 150 mm cell culture plate in serum depleted media for single biological repeat and this was done for 4 different passage number considering that to be 4 biological repeat) were diluted 20-fold to 250-fold and lysed with 0.5% Triton X100 (T9284-500ML; Merck Group, MA, USA). Absorbance was measured at 540 nm on a 96-well plate reader (352 Multiskan MS LabSystems Microplate Reader, 74165-5, Labsystem, Vantaa, Finland). Lipid content of EVs and NVEPs was measured using the sulfo-phospho-vanillin assay as previously described [28, 34]. Briefly, EV samples were treated with sulfuric acid, incubated at 90 °C, and subsequently reacted with phospho-vanillin reagent (ReagentPlus^®^, 99%, 79617, Merck, NJ, USA). The resulting colorimetric reaction was measured at 540 nm on a 96-well plate reader (352 Multiskan MS LabSystems Microplate Reader, 74165-5, Labsystem, Vantaa, Finland).

### Raman measurement

The Raman spectroscopy of EVs and NVEPs (seeding density of 5E + 05 cells per 150 mm cell culture) was performed by Dr. Jacopo Zini (Timegate Instruments, Oulu, Finland). In brief, the Timegate PicoRaman M3 (Timegate Instruments, Oulu, Finland) with a 100 ps 532 nm laser and complementary metal oxide single-photon avalanche diode was connected to an ÄKTA Pure 25 chromatography system via ProbeProMini (Timegate Instruments, Oulu, Finland) and SCHOTT ViewCell (Schott, Mainz, Germany). Raman spectra were measured continuously throughout the chromatography run with a 20-second acquisition time, 88 mW as the laser power, and a spot size of 100 μm.

### Lipidomic

For quantitative lipidomics, internal standards were added prior to lipid extraction. An amount of 100 µg protein measured by micro-BCA assay was subjected to lipid extraction from HEK293 cells, 100k-sEVs, and 167k-NVEPs were subjected to lipid extraction according to the protocol by Bligh and Dyer [35]. For single biological repeat all of them were pooled from nine 150 mm cell culture plates for 3 biological repeats. The analysis of lipids was performed by direct flow injection analysis (FIA) using a triple quadrupole mass spectrometer (FIA-MS/MS) and a high-resolution hybrid quadrupole-Orbitrap mass spectrometer (FIA-FTMS). FIA-MS/MS was performed in positive ion mode using the analytical setup and strategy described previously [36]. A fragment ion of m/z 184 was used for lysophosphatidylcholines (LPC) [37] The following neutral losses were applied: Phosphatidylethanolamine (PE) and lysophosphatidylethanolamine (LPE) 141, phosphatidylserine (PS) 185, phosphatidylglycerol (PG) 189, phosphatidic acid (PA) 115 and phosphatidylinositol (PI) 277 [38]. Sphingosine based ceramides (Cer) and hexosylceramides (Hex-Cer) were analyzed using a fragment ion of m/z 264 [39]. PE-based plasmalogens (PE P) were analyzed according to the principles described by Zemski-Berry [40]. Glycerophospholipid species annotation assumed of even numbered carbon chains only. A detailed description of the FIA-FTMS method was published recently [41]. Triglycerides (TG), diglycerides (DG) and cholesterol esters (CE) were recorded in positive ion mode m/z 500–1000 as [M+NH4] + at a target resolution of 140,000 (at 200 m/z). CE species were corrected for their species-specific response [42]. Phosphatidylcholines (PC), PC ether (PC O) and sphingomyelins (SM) were analyzed in negative ion mode m/z 520–960 as [M+HCOO]- at the same resolution setting. Analysis of free cholesterol (FC) was performed by multiplexed acquisition (MSX) of the [M+NH4] + of FC and the deuterated internal standard (FC[D7]) [42]. Lipid species were quantified using class-specific internal standards added prior to extraction. Molar percentages were calculated based on internal-standard–normalized peak areas, and a complete list of internal standards is provided in Supplementary Table T3.

LION-PCA heatmap analysis was performed. LION-PCA processes lipidomics datasets by scaling sum-normalized samples as z-scores (mean = 0, standard deviation = 1). PCA is then conducted, and loadings from a selected number of principal components (PrC) are used to rank lipid features. LION term enrichment is evaluated for all selected PrCs using the ranking mode with two-sided KS-tests, as previously described [43]. Significantly enriched LION-terms in any of the selected PrCs are identified. The associated lipid feature z-scores for these LION-terms are averaged per sample and visualized in a heatmap using the R package ‘pheatmap’ v1.0.12 [44]. Then we compared between 100k-sEV and 167k-NVEP by unpaired two tailed t-test and volcano plot to see enrichment of lipid species between them. Lipidomic enrichment analysis was conducted using LipidSig 2.0, a web-based bioinformatics platform for comprehensive lipid set enrichment and ontology analysis. Following untargeted lipidomic profiling, significantly regulated lipids were uploaded to LipidSig 2.0 for pathway and structural annotation. Enrichment was performed using default settings with lipid ontology categories based on class, subclass, saturation, and chain length.

### High-resolution flow cytometry measurement

All EV samples (which was isolated from three 150 mm cell culture plates in serum depleted media with seeding density of 5E + 06 cells per 150 mm cell culture plates) were analyzed using the Apogee A60 MP (SN 0130, Apogee Flow Systems, Northwood, UK) running Histogram Software v255.0.0.292 and FCM Control v 3.70. Prior to sample analysis, the system was cleaned using 1% bleach; sheath and diluent were checked to ensure machine and diluent cleanliness (event rate < 200 events/second). The Apogee platform was calibrated using a bead mixture (ApoCal 1524, Lot CAL0134, Apogee Flow Systems, Northwood, UK); monitoring beads were also analyzed (Apogee Mix 1527, Lot CAL0139, expiration 20-SEP-2021, Apogee Flow Systems, Northwood, UK) as part of daily protocols. Samples were analyzed for 60 s at a 3.01 µL/min flow rate, recording approximately 80,000 events per run. The analysis was performed using FlowJo 10.9.0.

### Super-resolution microscopy measurement

EV subtypes (14k-lEVs and 100k-sEVs) and 167k-NVEPs were isolated from cells cultured in serum-depleted medium. For each biological replicate, EV subtypes and NVEPs were isolated in parallel from three 150-mm culture plates seeded at identical densities (5 × 10⁶ cells per plate) and processed using the same isolation workflow. This procedure was repeated for three independent biological replicates. Isolated EVs/NVEPs were diluted tenfold in 1× PBS, deposited onto coverslips (VWR International, PA, USA), and incubated overnight at 4 °C in humidified chambers. Annexin V–Alexa Fluor 647 conjugate (Invitrogen, MA, USA; R37175) was added to the samples and incubated for 15 min at room temperature, protected from light.

For fixation, 4% PFA-PBS was used for 5 min at room temperature, followed by three washing steps of 5 min each with PBS. Antigen retrieval was carried out using 0.001% Triton X100 (T9284-500ML; Merck Group, MA, USA) in PBS for 5 min at room temperature, followed by 3 washing steps (3 × 5 min). Non-specific binding sites were blocked by 5% bovine serum albumin (BSA) in PBS at 37 °C for 35 min. All primary antibodies—anti-GFP (Catalog # A11122, Invitrogen, MA, USA), anti-HSP90β (Catalog # 37-9400, Invitrogen, MA, USA), and anti-CD9 (AF488), anti-CD81 (AF555), and anti-CD63 (AF647) antibodies (Unchained Labs, Pleasanton, CA, USA)—were prepared as specified in Table 1 and applied to the samples for 1 h incubation at room temperature, followed by three washing steps. The anti-CD9 (AF488; Catalog # UL039196), anti-CD81 (AF555; Catalog # UL041575), and anti-CD63 (AF647; Catalog # UL040751) antibodies were used according to the manufacturer’s instructions. Subsequently, Goat anti-Rabbit Alexa Fluor 568 (Catalog # A11011, Invitrogen, MA, USA) and Goat anti-Mouse Alexa Fluor 488 (Catalog # A11001, Invitrogen, MA, USA) secondary antibodies (Table 1) were applied for 1 h at room temperature, protected from light. Negative controls for dSTORM consisted of antibodies alone without EVs/NVEP to collect a background noise of unspecific antibody binding to the surface without EVs/NVEPs. Localization events obtained from antibody-only controls were quantified and used to define background thresholds, and signals below this threshold were excluded from clustering analysis.

After another washing step with sterile PBS (5 min), the cover glasses were placed on cavity slides (BR475505-50EA, Merck Group, MA, USA) filled with blinking buffer and sealed with two-component adhesive. Blinking buffer contained 100 U glucose oxidase (G2133-50KU, Merck Group, MA, USA), 2000 U CAT (C100, Merck Group, MA, USA), 55.56 mM glucose (G8270, Merck Group, MA, USA), and 100 mM cysteamine hydrochloride (M6500, Merck Group, MA, USA) in 1 mL final volume with sterile PBS. The dSTORM imaging was performed using a Nanoimager S (Oxford Nanoimaging ONI Ltd, Oxford, UK) instrument and evaluated by a built-in application of the corresponding quantification software CODI, called EV profiling, which identifies subpopulations with single EV particle using a customized clustering workflow shown in Fig. S5. Image acquisition was performed under highly inclined illumination using manufacturer-recommended laser settings, and localization reconstruction was carried out using ONI acquisition software prior to CODI-based clustering analysis. Cluster sizes were processed accounting for the size of primary antibodies with an average length of about 14 nm. All clustering parameters were kept constant across experimental and control conditions.

In addition to the procedure described above, we performed an extended dSTORM analysis to evaluate Hsp90β localization relative to CD63 under membrane-permeabilized and non-permeabilized conditions. EVs and 167k-NVEPs were labeled with anti-Hsp90β primary antibody (Table 1) preincubated with Alexa Fluor 568–conjugated secondary antibody (Table 1), together with CD63–Alexa Fluor 647 antibody (Leprechaun Human Exosome Kit, Unchained Labs) (Table 1). Samples were analyzed either in native conditions or following detergent treatment with 0.5% Triton X-100 (T9284-500ML; Merck Group, MA, USA) to assess luminal antigen accessibility. Mouse IgG (Table 1) preincubated with Alexa Fluor 568 secondary antibody was used as a negative control, and the resulting background signal was subtracted during quantitative analysis. Super-resolution image acquisition, reconstruction, and clustering analysis were performed using the same Nanoimager S platform and CODI EV profiling workflow described above.

### Functional uptake assay

HEK293T cells (for single passage cells were grown in 96 well plate with seeding density of 2.4E + 04 cells per well) were transfected with plasmids encoding EGFP-tagged versions of Arf6, CD63, LC3b, MysP, Rab7a, and soluble EGFP protein following the protocol detailed in the Cell Transfection section. The 167k-NVEPs were subsequently isolated from transfected cells as described in the Isolation of EVs and NVEPs section. Total protein content of each 167k-NVEP preparation was quantified using the micro-BCA assay (Micro BCA™ Protein Assay Kit, 500 mL per kit, Thermo Fisher Scientific, MA, USA). For the functional uptake assay, HEK293T cells seeded in 96 well plates were treated with increasing amounts of 167k-NVEPs—specifically, 1 µg, 5 µg, 20 µg, 100 µg, and 500 µg total protein per well. Cells were incubated with NVEPs for 6 h under standard culture conditions. Following incubation, cells were harvested and analyzed by flow cytometry to quantify the percentage of EGFP-positive cells (EGFP%). Measurements were performed using the CytoFLEX S N2-V3-B5-R3 Flow Cytometer (B78557, Beckman Coulter, Brea, USA) and CytExpert software (version 2.4.0.28, Beckman Coulter, Inc., Brea, USA).

### Statistical analysis

In this study, unpaired two-tailed t-tests and Mann-Whitney tests were conducted for comparison between two groups. For comparisons involving more than two groups, the dataset was initially assessed for normality and lognormality. Based on the results, either an ordinary one-way ANOVA with Tukey’s multiple comparisons test or the Kruskal-Wallis test was employed. For more than 3 groups we employed ordinary two-way ANOVA The analysis was performed utilizing GraphPad Prism 10 (GraphPad Software, La Jolla, USA). Deconvolution of the Raman spectroscopy was performed using OriginPro 2019 (OriginLab, Northampton, MA, USA), and other specialized software as appropriate. PCA was done using the Statistics and Machine Learning Toolbox™ of MATLAB version: 9.7.0.1190202 R2019b (MathWorks). PCA of both lipidomic and Raman data was done using singular value decomposition algorithm due to the number of variables exceeding the number of observations. Based on the Keiser method, the lipidomic data has only two meaningful components and the Raman data three, however, for the sake of easily interpretable visualization, the same parameters for both analyses were implemented – that is, the number of requested components was set to 3. Variables were centered prior to transforming the data into a reduced subspace. The code is available upon request.

## Results

### Distinct morphologies and surface markers define EVs and NVEPs

**Fig. 1:**
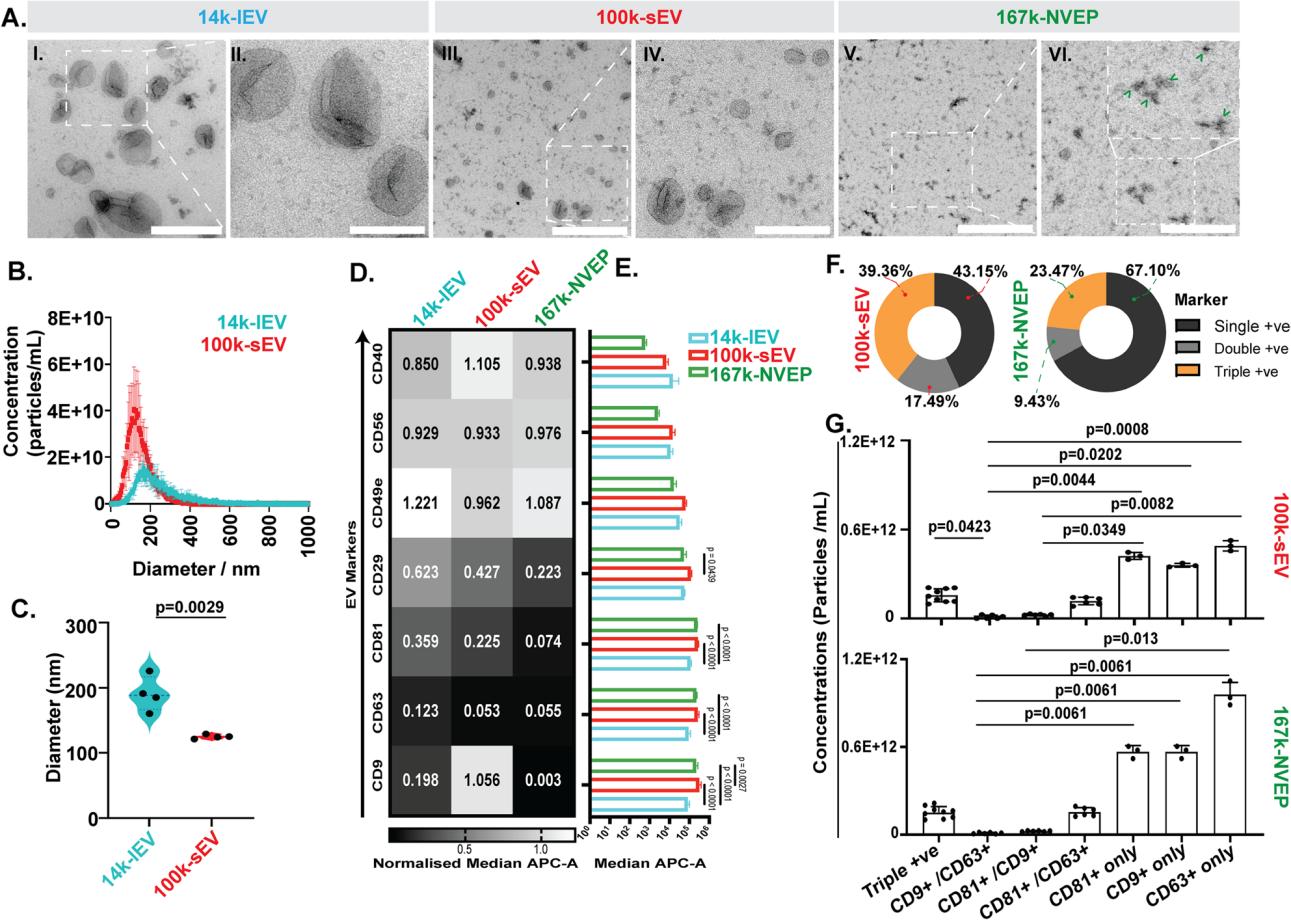
Characterization of size, surface charge, and marker enrichment in HEK293-derived EV subtypes and non-vesicular extracellular particles (NVEPs). **A** Transmission electron microscopy (TEM) images of 14k-IEV (complete field of view (FOV) - I, magnified - II), 100k-sEV (complete FOV - III, magnified - IV), and 167k-NVEP (complete FOV - V, magnified - VI) fractions derived from HEK293 cells. 14k-IEV and 100k-sEV fractions exhibit clear lipid bilayer-enclosed structures, while 167k-NVEP predominantly contains non-vesicular particles (green arrows). Scale bars: 500 nm (left panels), 200 nm (right panels). Images are representative of three biological replicates. **B** Nanoparticle tracking analysis (NTA) showing total mean concentrations of 5.29E+11 particles/mL for 14k-IEV and 9.60E+11 particles/mL for 100k-sEV fractions (n=4 biological replicates). **C** 14k-IEV (mean ± standard deviation diameter = 190.80 ± 26.87 nm) and 100k-sEV (mean ± standard deviation diameter = 125.20 ± 3.20 nm) fractions, with a statistically significant difference observed (*p*=0.0029, unpaired t-test; *n*=4 biological replicates). **D** Heatmap showing normalized median APC-A fluorescence intensities for seven EV surface markers (CD40, CD56, CD49e, CD29, CD81, CD63, CD9) detected on 14k-IEV (cyan), 100k-sEV (red), and 167k-NVEP (green) fractions. Values represent normalized median APC-A signal for each marker and EV subtype. **E** Quantitative bar graphs of median APC-A fluorescence intensity for each marker across the three EV subtypes, with statistical significance indicated (p-values from ordinary one-way ANOVA with Tukey’s multiple comparisons test). **F** Single-particle interferometric reflectance imaging sensing (SP-IRIS) analysis of vesicles positive for single (CD9+, CD63+, or CD81+), double (CD9+/CD63+, CD81+/CD9+, or CD81+/CD63+), and triple (CD9+, CD63+, and CD81+) markers in 100k-sEV and 167k-NVEP fractions. **G** 100k-sEV exhibited significantly higher expression of individual markers (*p*<0.05, Kruskal-Wallis test). A similar pattern was observed for 167k-NVEP, with CD63+ single markers predominantly expressed (*p*<0.05, Kruskal-Wallis test). Data represent mean ± standard deviation from at least three independent biological replicates

To understand the morphology and characteristics of EVs and NVEPs derived from HEK293 cells, we analyzed them with TEM which revealed their heterogenous morphology depending upon their size. EVs appeared spherical, cup-shaped, and varied in size, ranging from 41 to 849 nm with a visible lipid bilayer (Fig. 1A. I-IV) while 167k-NVEPs exhibited non-membranous structures (Fig. 1A. V, VI). Additionally, TEM of sectioned samples confirmed the presence of a lipid bilayer in both the 14k-lEV (Fig. S3 A) and 100k-sEV (Fig. S3 B) pellets, whereas the 167k-NVEP fraction displayed characteristic non-membranous, electron-dense structures (Fig. S3 C).

We determined the total concentration and size distribution of the 14k-lEVs and 100k-sEVs using NTA. There was no difference in the concentrations (Fig. 1B) and zeta potential (Fig. S4 B, C) between 14k-lEVs and 100k-sEV. However, 14k-lEV pellet was bigger than the 100k-sEV pellet (Fig. 1C). The size and zeta-potential individual particle distributions are shown in Fig. S4 C.

We assessed the surface markers of 14k-lEV, 100k-sEV, and 167k-NVEP using the MACSPlex Exosome Kit. Positive gating was established by comparing PE-A vs. APC-A signals against phosphate-buffered saline (PBS) controls, and markers with signal exceeding background were considered positive (Fig. S4 D). For each marker, the signal was first corrected by subtracting background from PBS and the highest negative isotype control signal (REA or mIgG1), resulting in the raw adjusted intensity. Each marker’s signal was divided by the average intensity of CD9, CD63, and CD81 to account for variations in marker abundance. This analysis revealed subtype-specific enrichment patterns: CD49e was highest in 14k-lEV, CD40 and CD9 were enriched in 100k-sEV, and CD49e also showed elevated levels in 167k-NVEP (Fig. 1D). Supplementary analysis further showed that CD146 was highly enriched in 14k-lEV and 100k-sEV, while most other markers showed minimal signals across subtypes (Fig. S4 E). The tetraspanins CD9, CD63, and CD81 had the highest absolute signal in 100k-sEV, consistent with their known enrichment in small EVs (Fig. 1E). In the supplementary dataset, CD146 and HLA-ABC were most abundant in 14k-lEV, while HLA-DRDPDQ showed the highest signal in 100k-sEV (Fig. S4 F).

Next, we used single-particle interferometric reflectance imaging sensor (SP-IRIS) to measure marker expression analysis of 100k-sEV and 167k-NVEP. Varying concentrations of single-positive (CD63+, CD9+, CD81+), double-positive (CD81+/CD63+, CD81+/CD9+, CD63+/CD9+), and triple-positive (CD81+/CD63+/CD9+) markers were observed in both 100k-sEVs and 167k-NVEPs (Fig. S4 G). Specifically, 100k-sEV showed 43.15% single positivity, 17.49% double positivity, and 39.36% triple positivity, while 167k-NVEPs had 67.1% single positivity, 9.43% double positivity, and 23.47% triple positivity (Fig. 1F). The concentration of 100k-sEVs expressing CD81+/CD63 + was significantly higher than that of 167k-NVEPs. Additionally, 100k-sEVs expressing both CD63 + and CD81 + were significantly more abundant than those expressing CD81+/CD9+. The concentration of 100k-sEVs expressing all three markers (CD63+, CD9+, CD81+) was also significantly higher than those expressing only CD63+/CD9+. The triple-positive EVs had a significantly higher concentration compared to the double-positive CD63+/CD9 + group. For 167k-NVEPs, the concentration of particles with all three markers were significantly higher than those expressing CD63+/CD9+, and the concentration of 100k-sEVs and 167k-NVEPs expressing CD63 + alone was significantly greater than those expressing CD81+/CD9+ (Fig. 1G).

The size distribution of tetraspanin-positive particles was higher than that of ApoB-positive particles in the 167k-NVEP pellet. Most tetraspanin-positive events were detected below 70 nm (Fig. S6 A). Diameter analysis showed that CD9-, CD63-, and CD81-positive 167k-NVEP particles exhibited significantly smaller sizes compared with ApoB-positive particles (Fig. S6 B). Quantification further showed that single-positive tetraspanin particles were present at higher concentrations than double-positive particles (Fig. S6 C). Co-localization analysis indicated that only *5E +* 0.15% of detected particles were ApoB/tetraspanin double positive (Fig. S6 D), suggesting that lipoprotein-associated particles represent a minor fraction of the 167k-NVEP preparation.

### NVEPs exhibit elevated protein-to-lipid ratios and unique raman signatures

**Fig. 2:**
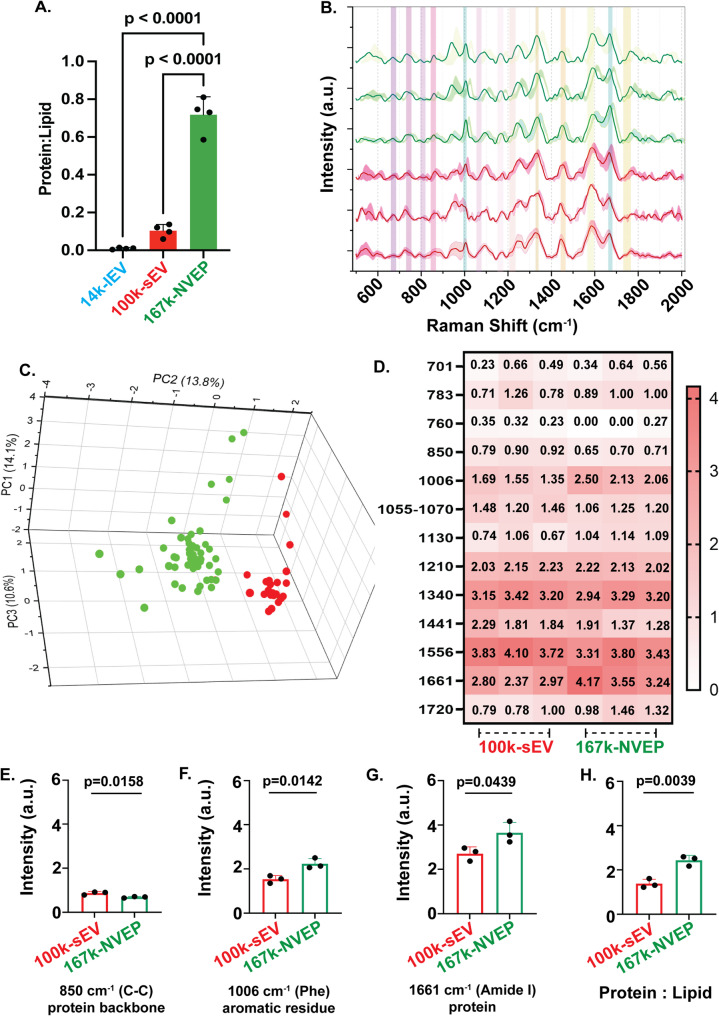
Biochemical characterization and Raman spectroscopy of EVs and non-vesicular extracellular particles (NVEPs). **A** Protein-to-lipid ratio is significantly higher in the 167k-NVEP fraction (0.72 ± 0.09) compared to both EV subtypes including 14k-lEV (0.0094 ± 0.0047) and 100k-sEV (0.103 ± 0.033) (*p* < 0.0001, Ordinary One-way ANOVA with Tukey’s multiple comparisons test). Data are presented as mean ± standard deviation (*n* = 4). **B** Raman spectroscopy of 100k-sEV (red) and 167k-NVEP (green) fractions, with solid lines representing average Raman spectra and shaded areas indicating standard deviation, showing shifts between 500 and 2000 cm− 1 (*n* = 3); the highlighted area are the representative Raman vibrational band assignments for HEK293-derived 100k-sEV and 167k-NVEP. **C** 3D scatter plot of Principal component analysis (PCA) showing cluster separation between 100k-sEV and 167k-NVEP fractions (*n* = 3). **D** Heat map of Raman spectral deconvolution showing key peaks in 100k-sEV and 167k-NVEP fractions. **E** The 850 cm− 1 band (C-C protein backbone, 0.87 ± 0.07 a.u) is predominant in 100k-sEV (*p* = 0.0158, unpaired t test), **F** while the 1006 cm− 1 (phenylalanine, 2.23 ± 0.23 a.u) and (**G**) 1661 cm− 1 (amide I, 3.65 ± 0.47 a.u) bands are enriched in 167k-NVEP (*p* = 0.0142 and *p* = 0.0439, respectively, unpaired t test). **H** Protein-to-lipid ratio in 167k-NVEP is significantly higher than in 100k-sEV (*p* = 0.0039, unpaired t test), which was compared between 1661 cm− 1 band (assigned as proteins) and 1441 cm− 1 band (assigned as saturated lipids) of Raman Fingerprint. Data are presented as mean ± standard deviation (*n* = 3)

The biochemical and label-free characterization of 14k-lEVs, 100k-sEVs, and 167k-NVEPs revealed distinct parameters that may serve as effective methods for distinguishing 167k-NVEP from EV subpopulations. The total lipid and protein contents of EVs and NVEPs were quantified by estimating the concentration, which was normalized per million cells seeded. The 167k-NVEP subpopulation exhibited significantly higher protein-to-lipid ratio compared to 100k-sEVs and 14k-lEVs, and in 100k-sEVs compared to 14k-lEVs (Fig. 2A). Raman spectroscopy was employed to distinguish the biochemical makeup between 100k-sEVs and 167k-NVEPs. While the overall spectral profiles of the 100k-sEV and 167k-NVEP populations appeared comparable, notable variations in peak intensities were observed. The Raman shifts recorded spanned from 500 to 2000 cm⁻¹ (Fig. 2B), with comprehensive peak assignments provided in the supplementary information (Fig. S7 A). To further distinguish the biochemical profiles, principal component analysis (PCA) was performed. Examination of the Raman spectra within the 500–2000 cm⁻¹ range revealed that the primary sources of variance were in the 924–1003 cm⁻¹ and 1274–1571 cm⁻¹ regions, corresponding to proteins, lipids, and nucleic acids, respectively. The main loading in principal component (PC)-1, which accounted for 14.1% of the variance and was the key factor differentiating 100k-sEVs from 167k-NVEPs, was detected at 1006 (assigned to phenylalanine, Phe), 1661 (assigned as Amide I), and 1441 cm⁻¹ (assigned as saturated lipid), corresponding to proteins and lipids. A 3D PCA score plot constructed using PC1 (14.1%), PC2 (13.8%), and PC3 (10.6%), exhibited a 99% separation between the two populations (Fig. 2C). A heat map of the Max Height for specific Raman shifts, including 1006 cm⁻¹ (assigned to phenylalanine, Phe) and 1661 cm⁻¹ (assigned to Amide I, proteins), showed that these Raman fingerprints were predominantly enriched in 167k-NVEPs compared to 100k-sEVs (Fig. 2D). This enrichment indicates a biochemical distinction in protein content between the two populations, particularly for protein structures represented by phenylalanine side chains and the Amide I band. At 850 cm⁻¹, representing C-C bonds (assigned to the protein backbone), 100k-sEVs showed significantly higher levels compared to 167k-NVEPs (Fig. 2E). In contrast, Raman shifts at 1006 cm⁻¹ (assigned to phenylalanine, an aromatic residue of proteins) (Fig. 2F) and 1661 cm⁻¹ (assigned to amide I, proteins) were significantly higher in 167k-NVEP (Fig. 2G). No significant differences were observed at other Raman shifts (Fig. S7 B). Notably, the analysis revealed that the 167k-NVEP population exhibited a significantly higher protein-to-lipid intensity ratio compared to 100k-sEV (Fig. 2H), suggesting that 167k-NVEP is characterized by an enrichment in proteins relative to lipids. This distinction underscores the unique biochemical properties of 167k-NVEPs in comparison to 100k-sEVs.

### Lipidomic profiling reveals distinct lipid signatures in NVEPs

**Fig. 3:**
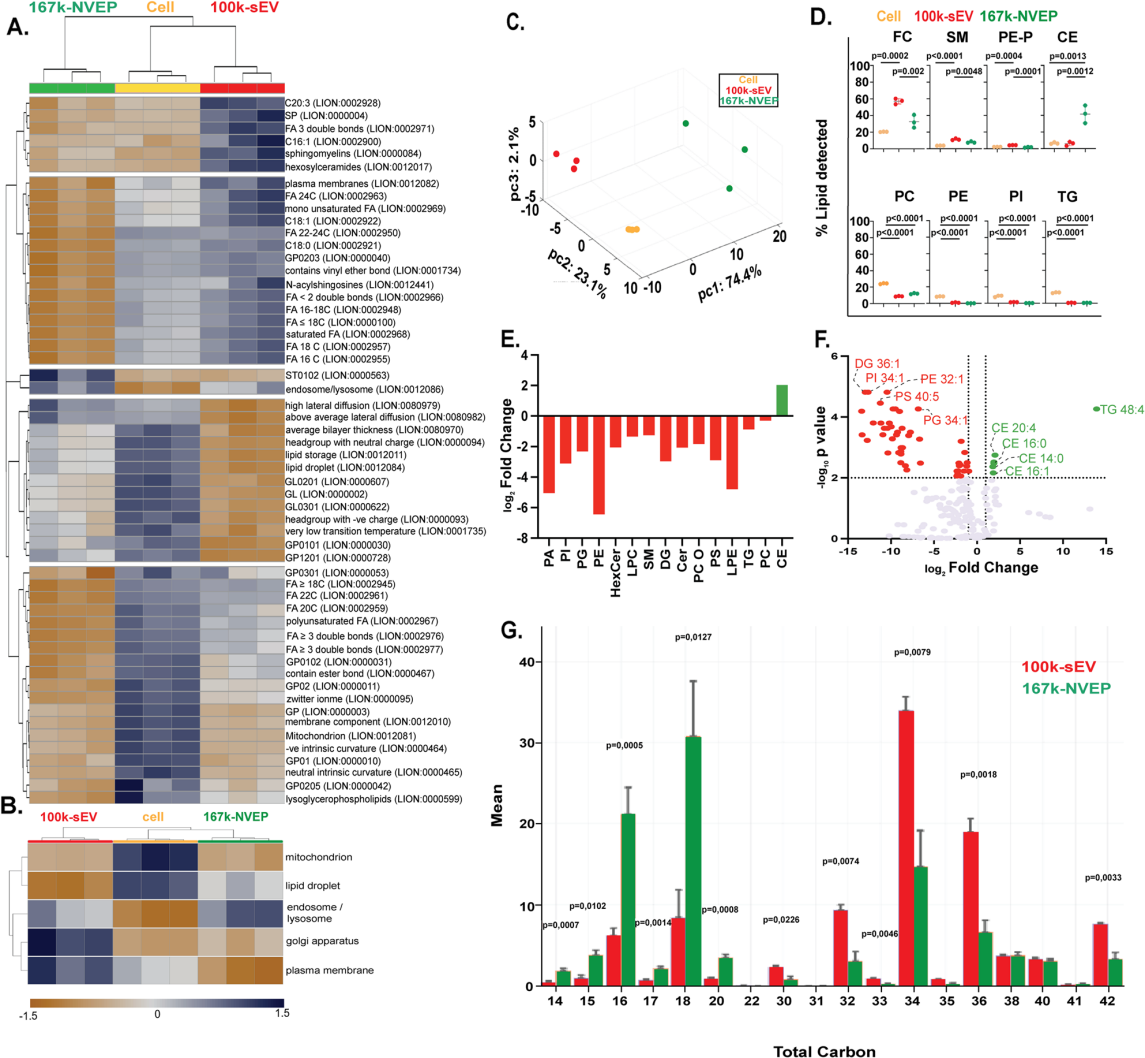
Lipidomic profiling and LION-term enrichment analysis of HEK293 cells, 100k-sEV, and 167k-NVEP. **A** Heatmap (z-scores: yellow, < 0; blue, > 0) from subcellular lipidomic data for cells, 100k-sEV, and 167k-NVEP. **B** Lipids in 167k-NVEP are primarily linked to endosome/lysosome compartments, while 100k-sEV lipids are associated with Golgi apparatus, and plasma membrane. **C** Three-dimensional principal component analysis (PCA) plot displaying the distribution of cells (yellow), 100k-sEV (red), and 167k-NVEP (green) based on molecular signatures. Each point represents an independent biological replicate (*n*=3). **D** 100k-sEV is predominantly enriched in sphingomyelin (SM), and phosphatidylethanolamine-based plasmalogen (PE-P), whereas 167k-NVEP is highly enriched in cholesteryl ester (CE). **E** Bar graph illustrating log₂ fold change in abundance of major lipid classes between 100k-sEV (red) and 167k-NVEP (green). Most lipid species, including phosphatidic acid (PA), PI, phosphatidylglycerol (PG), PE, hexosylceramide (HexCer), lysophosphatidylcholine (LPC), SM, diacylglycerol (DG), ceramide (Cer), PC, phosphatidylserine (PS), lysophosphatidylethanolamine (LPE), and TG, are significantly depleted in 100k-sEV relative to 167k-NVEP, while CE is enriched in 167k-NVEP. **F** Volcano plot depicting lipids significantly enriched in 100k-sEV (red) include DG 36:1, PI 34:1, PE 32:1, PS 40:5, and PG 34:1. Lipids significantly enriched in 167k-NVEP (green) include TG 48:4 and CE (CE 20:4, CE 16:0, CE 14:0, CE 16:1). Dotted lines indicate thresholds for statistical significance (*p*<0.01) and fold change. All data represents mean values from three independent biological replicates (*n*=3). **G** The 167k-NVEP fraction (green bars) shows significant enrichment in lower carbon chain lipids, while 100k-sEV (red bars) exhibits selective enrichment at longer chain lengths. Data represent mean relative abundance ± standard deviation from n=3 biological replicates, with statistical significance determined by unpaired t-tests (*p*<0.001, *p*<0.01). Findings suggest distinct lipid species between EV subtypes and 167k-NVEPs

To characterize the lipid composition of 167k-NVEPs and compare it to 100k-sEV and secreting cells, we conducted lipidomic analysis using LION and LipidSig 2.0 [27–29]. Hierarchical clustering heat maps which include lipid classification, chemical and physical properties (fatty acid length and unsaturation, headgroup charge, intrinsic curvature, membrane fluidity, bilayer thickness), function, and sub-cellular component (predominant sub-cellular localization) illustrate significant lipidomic variations between 100k-sEVs and 167k-NVEPs (Fig. 3A, B). 100k-sEV exhibited higher concentrations of sphingomyelins (SM), hexosylceramides (HexCer), and unsaturated fatty acids, while 167k-NVEP showed significant upregulated of ST0102 represents steryl esters (Fig. 3A). Secondly, to infer the potential subcellular origin of the lipid cargo in 100k-sEVs and 167k-NVEPs, we performed compartment-based lipid enrichment analysis. This revealed that 167k-NVEPs were enriched in lipid species typically associated with the endosome–lysosome system and lipid droplets, whereas 100k-sEVs showed higher abundance of lipids linked to the Golgi apparatus (Fig. 3B). To examine how lipids are differentially distributed from the secreting cell into specific EV subtypes, we conducted PCA on the lipidomic profiles of parent HEK293 cells, 100k-sEVs, and 167k-NVEPs. PCA demonstrated clear separation of the three sample types along PC1 (74.4%), with the 100k-sEVs and the 167k-NVEPs grouping separately from cells (Fig. 3C). This distinction is also evident in the PCA of specific EV-related lipid classes, including free cholesterol (FC), SM, phosphatidylethanolamine plasmalogen (PE-P), phosphatidylethanolamine plasmalogen (PE-P), cholesteryl esters (CE), phosphatidylcholine (PC), phosphatidylethanolamine (PE), phosphatidylinositol (PI), and triglycerides (TG) (Fig. S8 A).

Quantitative lipidomic analysis revealed distinct lipid class compositions across cells, 100k-sEVs, and 167k-NVEPs. Phosphatidylcholines accounted for approximately 28–29 mol% of total lipids in cells, 12–13 mol% in 100k-sEVs, and 14–15 mol% in 167k-NVEPs, while sphingomyelins were relatively enriched in EV fractions compared with cells (Supplementary Table T4). All detected lipid classes and their corresponding molar percentages are provided in the Supplementary Information.

Further analysis of individual lipid classes demonstrated a predominance of cholesteryl esters (CE) in the 167k-NVEP fraction compared with both 100k-sEVs and secreting cells, whereas 100k-sEVs exhibited higher relative abundances of free cholesterol (FC), sphingomyelins (SM), and plasmalogen phosphatidylethanolamines (PE-P). In contrast, secreting cells were enriched in phosphatidylcholines (PC), phosphatidylethanolamines (PE), phosphatidylinositols (PI), and triacylglycerols (TG) (Fig. 3D). In addition, although most lipid species were more abundant in cells, 100k-sEVs showed notably higher relative levels of plasmalogen phosphatidylcholines (PC-O) and phosphatidic acid (PA), lipid classes implicated in membrane dynamics and vesicle formation (Fig. S8 B). These findings align with previous research indicating that EVs possess distinct lipid profiles, which can impact their structural characteristics and functional roles [30, 31]. LipidSig 2.0-based enrichment analysis confirmed compositional distinctions: CE was elevated in 167k-NVEPs (Fig. 3e) while the volcano plot shows triglyceride 48:4 and CE species (20:4, 16:1, 16:0, 14:0) as NVEP-enriched. Conversely, the top five lipids enriched in 100k-sEVs were diacylglycerol 36:1, PI 34:1, PE 32:1, phosphatidylserine (PS) 40:5, and phosphatidylglycerol (PG) 34:1 (Fig. 3F). Notably, carbon chain length distributions diverged significantly: 167k-NVEPs exhibited higher abundance of 16-, 18-, and 20-carbon chains, while 100k-sEVs were enriched in 32-, 34-, 36-, and 42-carbon chains (Fig. 3G).

### Membrane association quantifies marker biogenesis pathways

**Fig. 4:**
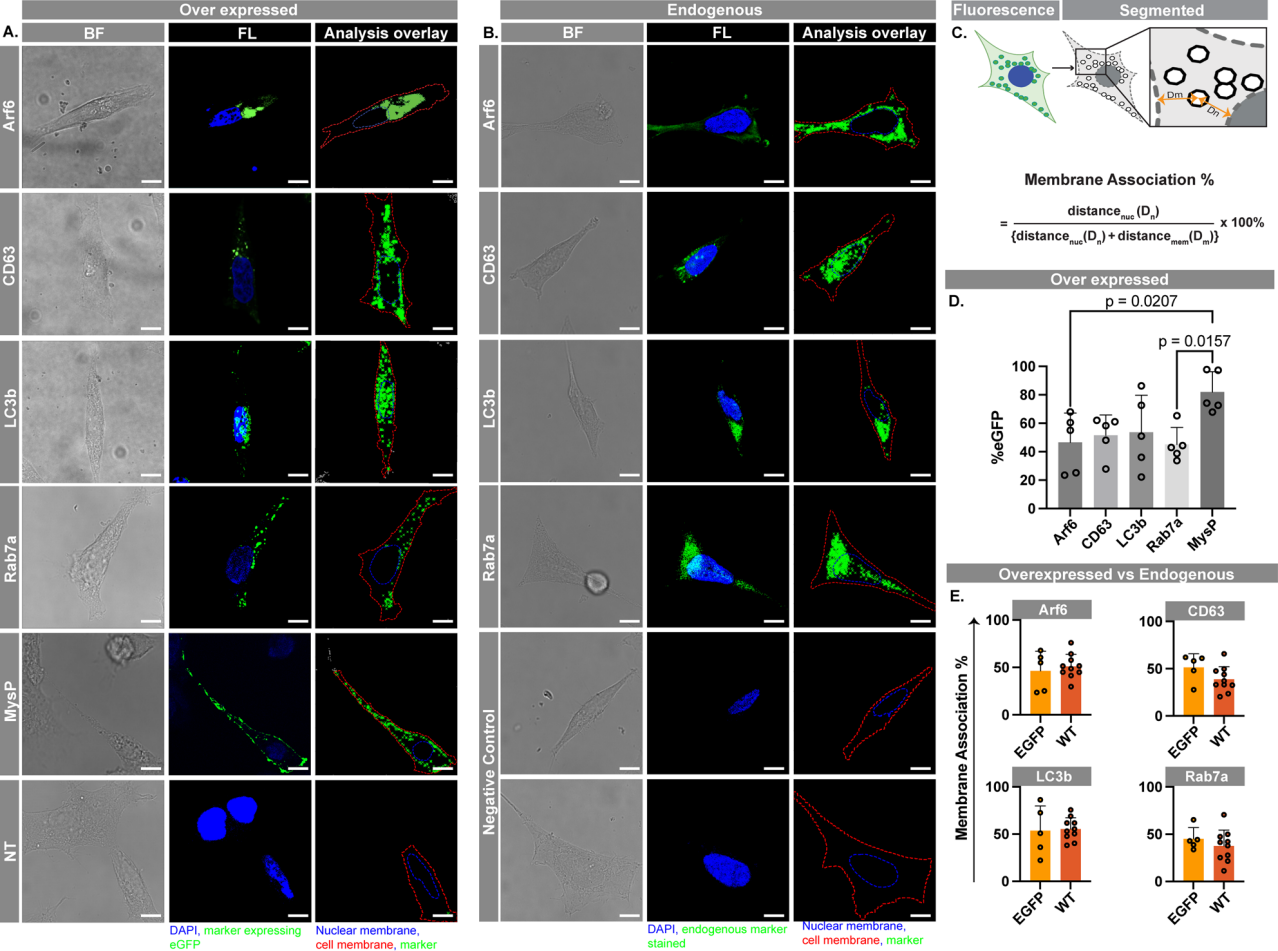
Subcellular localization and membrane association of EV biogenesis markers in HEK293 cells. **A** Subcellular localization of overexpressed EV biogenesis markers in HEK293 cells. Cells expressing EGFP-tagged ADP-ribosylation factor 6 (Arf6), CD63, microtubule-associated protein 1 light chain 3 beta (LC3B), Myristoylation/Palmitoylation signal peptide (MysP), and Rab GTPase 7a (Rab7a) were imaged by confocal microscopy. Bright-field (BF) and fluorescence (FL) micrographs were used to segment the nucleus, plasma membrane, and EGFP signal (analysis overlay). Scale bar, 10 μm. **B** Subcellular localization of endogenous EV markers. Cells were immunostained for Arf6, CD63, LC3B, MysP, and Rab7a and imaged under confocal microscopy. BF and fluorescence images were used to segment the nucleus, plasma membrane, and endogenous fluorescence signals (analysis overlay). Scale bar, 10 μm. **C** Schematic representation of membrane association analysis. We calculated the shortest distance between segmented fluorescence signals and the plasma membrane and nucleus to derive the membrane association percentage, which reflects relative proximity of the signal to the plasma membrane. **D** Quantitative comparison of membrane association for overexpressed markers. MysP served as a membrane-associated reference (81.91 ± 14.40%). CD63 (51.46 ± 14.36%) and LC3B (53.62 ± 26.13%) did not differ significantly from MysP (*p* > 0.05), whereas Arf6 (46.46 ± 20.70%, *p* = 0.0250) and Rab7a (45.01 ± 12.07%, *p* = 0.0184) showed reduced membrane association. Statistical analysis used ordinary one-way ANOVA followed by Dunnett’s multiple comparisons test. **E** Comparison of membrane association between overexpressed (EGFP) and endogenous (WT) proteins. Membrane association values did not differ significantly between endogenous and overexpressed conditions for Arf6 (51.11 ± 12.77%), CD63 (38.70 ± 13.47%), LC3B (55.30 ± 11.95%), or Rab7a (37.52 ± 16.90%), as determined by ordinary one-way ANOVA with Dunnett’s multiple comparisons test

To trace the biogenesis route of EV subtypes and 167k-NVEP, we expressed EGFP-tagged markers (Arf6, CD63, LC3B, MysP, and Rab7a) in HEK293 cells and developed a quantitative membrane association assay. We segmented confocal micrographs to define nuclei and plasma membranes and calculated, for each fluorescence signal, the minimum distance to these compartments to derive a membrane association percentage (Fig. 4A; analysis workflow shown in Fig. 4C). This approach enabled quantitative comparison of membrane proximity among markers using MysP as a membrane-associated reference.

Analysis of confocal images from cells expressing these markers (*n* = 5) indicated that Rab7a, and Arf6 showed significantly lower membrane association than MysP (Fig. 4D). The reduced membrane association of Rab7a, and Arf6 implies that these markers are primarily located within endosomal compartments rather than at the plasma membrane, which is consistent with their known functions in ILV formation and endocytosis regulation [32]. Autophagosome marker LC3b exhibited levels of membrane association comparable to MysP, with no statistically significant difference noted. This localization aligns with previous studies describing the interaction of LC3b with membrane-associated autophagic structures (34). Furthermore, the tetraspanin protein CD63, known for its role in exosome biogenesis, did not show significant differences in membrane association compared to MysP, suggesting a distribution across both endosomal and plasma membrane locations. In contrast, Arf6, which is also associated with vesicle trafficking closer to the nucleus, displayed a distinct pattern in this study. This observation is in line with earlier reports highlighting the role of Arf6 in nuclear-proximal vesicle transport [32].

To exclude potential artefacts caused by EGFP tagging or protein overexpression, we next examined the localization of the corresponding endogenous proteins by immunofluorescence under identical imaging and analysis conditions (Fig. 4B). Quantitative comparison revealed no significant differences in membrane association between endogenous proteins and their EGFP-tagged counterparts (Fig. 4E), indicating that overexpression and EGFP fusion did not measurably alter subcellular localization in this system.

### Comprehensive characterization of EV and NVEP heterogeneity

EV subtypes and 167k-NVEPs were isolated from HEK293 cells expressing selected markers for characterization as well as from non-transfected (NT) control conditions. NTA was used to determine concentration and size of 14k-lEV and 100k-sEVs (Fig. S9 A-D). Total lipid content was significantly higher in 14k-lEVs than in 100k-sEVs or 167k-NVEPs (Fig. S9 E). Conversely, 167k-NVEPs exhibited significantly higher protein content than either 14k-lEVs or 100k-sEVs (Fig. S9 F). Critically, the protein-to-lipid ratio of 167k-NVEPs was significantly higher than that of all EV subtypes, consistent across both NT and overexpressing conditions (Fig. S9 G). Following the Minimum Information for Flow Cytometry studies of Extracellular Vesicles (MIFlowCyt-EV) guideline [34], we estimated the EGFP% and concentration (events/mL) of the over-expressed EV subtypes, where gating was made in respect to their NT counter parts (Fig. S10 A). Histogram showing the comparison between the marker expressing EV subtypes and their NT counterparts (Fig. S10 B). No markers showed any significant difference in the EGFP% between their EV subtypes (Fig. S10 C), whereas LC3b and MysP expressing 14klEV events/mL were significantly higher than their 100k-sEV counterparts (Fig. S10 D). Conversely, Rab7a expressing 100k-sEV events/mL was significantly higher than 14k-lEV. Taken together, we found that no marker is exclusive in either EV subtypes, but it is possible to see an enrichment in CD63 and MysP for 14k-lEV or CD63 and Rab7a for 100k-sEV (Fig. S10 E).

**Fig. 5:**
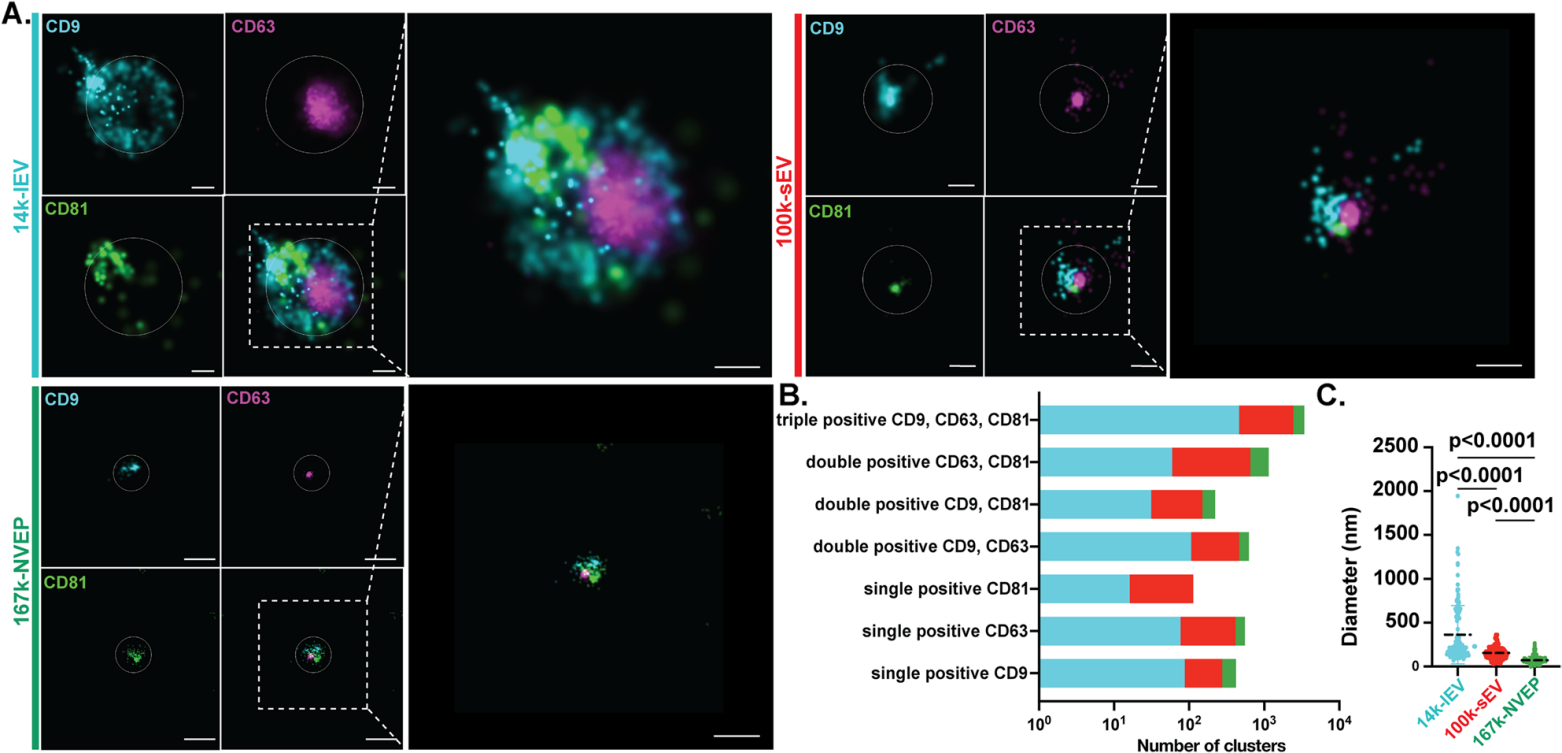
Super-resolution profiling of tetraspanin phenotypes and size distribution across EV subtypes and 167k-NVEP populations. **A** Super-resolution characterization of native EVs and 167k-NVEPs by direct Stochastic Optical Reconstruction Microscopy (dSTORM). Representative dSTORM images of immunolabeled CD9, CD63, and CD81 were acquired for both native EVs and NVEPs. Particles were stained with fluorophore-conjugated antibodies against the tetraspanins CD9, CD63, and CD81. Scale bars, 100 nm. **B** Quantification of cluster compositions showing the number of single-, double-, and triple-positive tetraspanin clusters in 14k-lEV (cyan), 100k-sEV (red), and 167k-NVEP (green). Statistical analysis was performed using Ordinary two-way ANOVA. **C** Scatter plot displaying the diameter distribution of individual particles across 14k-lEV, 100k-sEV, and 167k-NVEP populations. Mean values are indicated by horizontal dashed lines (*p*<0.0001, Kruskal–Wallis test)

In addition to these measurements, we employed direct stochastic optical reconstruction microscopy (dSTORM) to perform single-particle analysis of 14k-lEVs, 100k-sEVs, and 167k-NVEPs isolated in parallel from around thirty million producer cells, enabling quantitative assessment of marker-positive particles. Firstly, we checked the distribution of the known canonical markers of the EVs including CD9, CD63 and CD81 across NT EV subtypes and 167k-NVEP (Fig. 5A), where we observed that 100k-sEV triple positivity clusters were significantly higher than 14k-lEV and 167k-NVEP (Fig. 5B). In terms of size, 14klEV was larger followed by 100k-sEV and 167k-NVEP (Fig. 5C).

For the marker expressing EV subtypes, we used Annexin V as a membrane marker due to its phosphatidylserine-binding capability, along with an anti-GFP antibody targeting EGFP-tagged markers previously studied. For 167k-NVEPs, which lack a membrane for Annexin V binding, we utilized the reported marker HSP90ß [8, 35], as 167k-NVEP single positive annexin-V clusters was significantly lower than 100k-sEV (Fig. S5 A). A representative image shows the cluster finding by applying the EV profiling in the CODI webtool (Fig. S5 B).

**Fig. 6:**
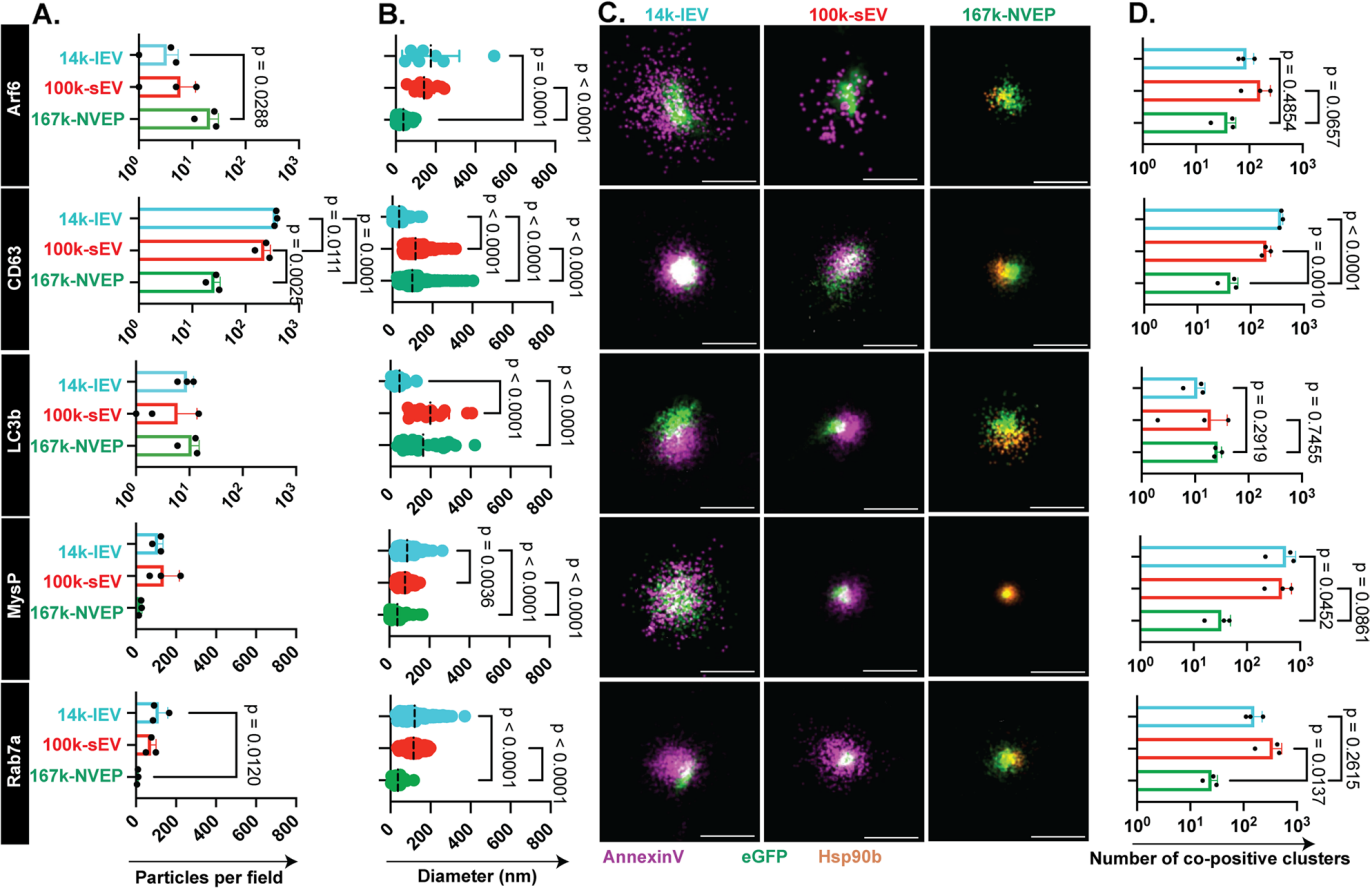
Marker expression profiles, size distribution, and colocalization phenotypes in EV and 167k-NVEP populations revealed by super-resolution imaging. **A **Bar plots represent the number of single EVs and NVEPs per field expressing the indicated markers—Arf6, CD63, LC3b, MysP, and Rab7a —detected in 14k-lEVs (cyan), 100k-sEVs (red), and 167k-NEVPs (green). Across biological replicates (*n*=3), 167k-NEVPs exhibited significantly higher numbers of Arf6-positive particles per field compared to 14k-lEVs (p=0.0288, Ordinary one-way ANOVA with Tukey's multiple comparisons test). Similarly, significant differences were observed in the expression patterns of other markers, including higher CD63 compared to 100ksEV (p=0.0111, Ordinary one-way ANOVA with Tukey's multiple comparisons test) and compared to 167k-NEVPs (p=0.0001, Ordinary one-way ANOVA with Tukey's multiple comparisons test ) and significant increase of Rab7a expressing 14k-lEVs compared to 167k-NEVPs (p = 0.0102, Ordinary one-way ANOVA with Tukey's multiple comparisons test). **B** Diameter measurements of individual particles show that 14k-lEVs were significantly larger than both 100k-sEVs and 167k-NEVPs, and that 100k-sEVs were significantly larger than 167k-NEVPs. **C** Representative super-resolution images of single EV and NVEP particles with their respective markers. 14k-lEVs and 100k-sEVs were labeled with Annexin V (magenta) and anti-EGFP antibody (green), while 167k-NEVPs were stained with anti-HSP90β (orange) and anti-EGFP (green). Scale bar: 100 nm. **D** Population Phenotypes of the double positive clusters shown where co-positive cluster of HSP90β and CD63 expressing 167k-NVEP was significantly lower than EV counterparts and similar findings were observed for the co-positive cluster of MysP and Rab7a expressing 167k-NVEPs

We evaluated diameters for all markers expressing EV subtypes and 167k-NVEP samples expressing the selected markers, comparing these parameters across different groups. NT EV subtypes and NVEP fractions were used during dSTORM assay optimization to define baseline antibody staining and signal-to-noise characteristics; therefore, only engineered vesicle populations are presented in the comparative analyses. CD63-expressing 14k-lEVs and 100k-sEVs had significantly higher particle counts than 167k-NVEPs, while Arf6-expressing 167k-NVEPs exceeded 14k-lEVs in particles per field (Fig. 6A). The diameter measurements of 14k-lEVs were consistently larger than those of 100k-sEVs and 167k-NVEPs (Fig. 6B). Representative EVs and NVEPs for all the markers used in this study were shown in Fig. 6C. Our data revealed distinct marker abundances across subpopulations. Quantitative co-localization analysis using Annexin V (for 14k-lEVs and 100k-sEVs) and HSP90β (for 167k-NVEPs) with various markers revealed distinct population phenotypes among double-positive clusters. The fraction of HSP90β and CD63 co-expressing particles in 167k-NVEPs was significantly reduced compared to EV populations, with similar reductions observed for MysP and Rab7a co-positive clusters within the 167k-NVEP fraction (Fig. 6D). None of the markers used in this study seem to be exclusive for any EV subtypes or 167k-NVEPs. Interestingly, 14k-lEV was more abundant with CD63, MysP (Fig. S11 A); 100k-sEV was more abundant with CD63, Rab7a (Fig. S11 B) and 167k-NVEP was more abundant with Arf6 and CD63 (Fig. S11 C). To assess the consistency of size measurements across orthogonal platforms, we compared the particle diameters obtained from dSTORM with those measured by NTA. Many diameters were consistent across platforms except for CD63, MysP and Rab7a expressing 14k-lEV as well as CD63 and MysP expressing 100k-sEV being significantly larger by NTA than dSTORM (Fig. S12 A, B). Finally, we assessed if 167k-NVEPs can deliver EGFP to recipient cells. Different protein amounts (1 µg, 5 µg, 20 µg, 100 µg, 500 µg) were added to native HEK293 cells (Fig. S13 A), where we observed that CD63 expressing 167k-NVEPs had the highest efficiency of EGFP transfer reaching 54.60% of treated cells becoming EGFP positive (Fig. S13 B).

Lastly, dSTORM analysis showed that detergent treatment markedly reduced the CD63 signal in 100k-sEVs, whereas CD63-associated signals persisted in the 167k-NVEP fraction (Fig. S14 A). Corresponding changes in cluster diameter are shown in Fig. S14 B. Immuno-electron microscopy further confirmed CD63 labeling in the classical 100k-sEV pellet (Fig. S14 C) and, notably, also within the 167k-NVEP pellet (Fig. S14 D).

## Discussion

Here in this study, we have used HEK293 cell-derived EVs and NVEPs. This is due to the unique advantages of HEK293 for EV and NVEP research, as substantiated by leading studies in the field [13, 45, 46]. HEK293 cells are human-derived, highly amenable to genetic engineering, and capable of robust, scalable, and serum-free EV production—enabling precise manipulation of EV surface features and cargo for mechanistic studies and therapeutic development. These attributes ensure consistent particle composition and purity and minimize batch variability. Despite these advantages that enabled our comprehensive multi-parametric characterization of EVs and NVEPs, we believe future work with other cell types or biofluids would be highly valuable. Differential ultracentrifugation remains the most widely adopted method for EV isolation, accounting for over 50% of all EV separations in current research practice as documented in the EV-TRACK knowledgebase [47]. While density gradient ultracentrifugation, size-exclusion chromatography and asymmetric flow field-flow fractionation provide enhanced purity for EV isolation from cell culture supernatants, these methods are associated with significantly reduced yields, extended processing times, and increased technical complexity that limit their practical application for large-scale comparative studies, whereas differential centrifugation with filtration offers superior throughput, cost-effectiveness, and sufficient separation efficiency for comprehensive characterization of EV subpopulations from conditioned media [48]. Our stepwise protocol (14,000 ×g, 100,000 ×g, 167,000 ×g) with complementary filtration represents an optimized balance between isolation efficiency and practical feasibility, ensuring sufficient material recovery for multimodal characterization across biochemical, lipidomic, and functional platforms. While specific nomenclatures such as ‘exomeres’ and ‘supermeres’ have been proposed for distinct non-vesicular nanoparticle subtypes, the current paucity of comprehensive comparative studies and the substantial research required to definitively distinguish these particles necessitate a more inclusive terminology. Therefore, we designate our 167,000×g pellet as ‘167k-NVEPs’ to acknowledge both the operational definition based on isolation parameters and the need for extensive future investigations to fully characterize the heterogeneity within this non-vesicular particle population.

To have better understanding the NVEPs composition and source of origin, we employed orthogonal techniques to compare EV subpopulations to NVEPs including TEM, NTA, zeta potential measurement, multiplexed-bead assay and high-resolution flow cytometry, SP-IRIS, biochemical assays, Raman spectroscopy, lipidomic, and super-resolution microscopy. TEM images confirmed the presence and absence of lipid bilayer membranes in EV subpopulations (14k-lEV and 100k-sEV) and 167k-NVEP [8], respectively. This morphological contrast aligns with previous reports describing exomeres, a type of NVEP, as non-membranous entities. Across our NVEP populations, canonical EV markers were nevertheless detected. Notably, Zhang et al. reported that supermeres lack CD9, CD63, and CD81 expression [8], and Noguchi et al. found BeWo cell–derived exomeres to be positive for AGO2 but negative for CD63, CD9, CD81, FLOT1, and TSG101 [49]. The presence of these tetraspanins in our 167k-NVEPs may therefore reflect cell type–specific expression or variations in isolation parameters.

Interestingly, we observed that NVEP exhibited high protein to lipid ratio, which can readily distinguish and characterize through this straightforward, reliable, reproducible, and cost-effective method. Characteristic bands corresponding to major EV components were observed in the fingerprint region of Raman spectra for all EV samples, consistent with previous findings [50, 51]. Our Raman spectroscopy results showed a significantly high protein-to-lipid ratio of 167k-NVEP, aligning with our biochemical analysis data. However, prolonged ultracentrifugation at high centrifugal forces can promote co-sedimentation of soluble proteins and formation of protein aggregates, which may contribute to the compositional features observed in the 167k-NVEP fraction. Consistent with this interpretation, single-particle fluorescence analysis detected ApoB-positive particles in only a small proportion (~ 0.15%) of the 167k-NVEP fraction, indicating limited contribution from lipoprotein-associated particles. Additional Raman spectroscopy should be conducted to better understand the C-H Stretching (2800–3000 cm⁻¹) region, which can be further correlated with lipidomic data to elucidate characteristic differences between 167k-NVEPs and 100k-sEVs.

Lipidomic profiling revealed 167k-NVEPs as a biochemically distinct extracellular nanoparticle population, enriched in steryl esters and CE compared to 100k-sEVs [52, 53]. Previous work by Emanuel et al. [54] demonstrated that exomeres are characteristically enriched in cholesterol, and our lipidomic profiling extends this observation by revealing that the 167k-NVEP fraction not only contains elevated cholesteryl esters—particularly CE 20:4, CE 16:0, CE 14:0, and CE 16:1—but also shows significant enrichment of triglyceride TG 48:4, underscoring a unique lipid signature that may inform their biogenesis and functional properties. The lipidomic analysis reveals that 167k-NVEPs possess a lipid composition sharply distinct from both 100k-sEVs and their parent cells, underscoring NVEPs as a discrete class of extracellular particles with unique biophysical properties and likely specialized functions. 100k-sEV were found to contain lipids associated with intercellular communication, though certain enriched lipid species suggest the inclusion of additional structures, potentially from amphisome [55]. The 100k-sEV fraction also showed an abundance of sphingolipids, including sphingomyelins (SM) and hexosylceramides (HexCer), which are essential for exosome formation and play roles in brain cell function [56, 57]. A previous study showed that SM and HexCer which are enriched in 100k-sEV can support membrane structure and promote amyloid-β clearance through exosome secretion in microglia, suggesting neuroprotective roles for these EVs [58]. The predominant sub‑cellular localization assigned by LION analysis reflects enrichment of characteristic lipid classes and does not necessarily recapitulate the exact biogenetic route of the vesicles. While 100k‑sEVs are of endosomal origin, their lipid composition is strongly shaped by biosynthetic pathways emanating from the Golgi apparatus. Specifically, the enrichment of Golgi‑associated lipids in 100k‑sEVs is consistent with models of exosome biogenesis in which lipid fluxes from the trans‑Golgi network—particularly sphingomyelin (SM) and other complex lipids—are directed to the multivesicular body (MVB) limiting membrane prior to intraluminal vesicle formation. This direct flux of lipids from the Golgi to the exosomal pathway is well documented [59] and provides a mechanistic basis for the Golgi‑like lipid signature of endosomally derived exosomes. In contrast, the enrichment of endosome/lysosome‑ and lipid droplet‑associated lipids in 167k‑NVEPs points to a distinct biogenetic input, likely involving direct recruitment from late endosomal or lipid storage compartments, thereby distinguishing these particles from the canonical exosomal pathway. Notably, prior work using AF4-purified exomeres demonstrated a relative enrichment of ceramide species compared to sphingomyelins in canonical EV fractions, underscoring a distinctive lipid profile for non-vesicular nanoparticles [8]. The prevalence of esterified sterols implies altered membrane rigidity and fusion dynamics, raising the possibility that NVEPs serve specialized functions in hydrophobic cargo transport or modulation of recipient cell lipid metabolism. This distinct lipid signature suggests a non-endosomal, possibly secretory lysosomal origin, consistent with previous observations that exomeres may arise via alternative non-canonical secretion pathways [29, 60].

The enrichment of neutral lipids (CE and TG) in 167k-NVEPs raises the question of how such hydrophobic cargo is stabilized in the extracellular space. Unlike bilayer-enclosed EVs, where neutral lipids would disrupt membrane integrity, our data support a lipoprotein-like or lipid-droplet-like architecture for NVEPs. In this model, a hydrophobic neutral lipid core is shielded by a phospholipid monolayer and a dense protein shell, consistent with the observed high protein-to-lipid ratio, negligible Annexin V binding (absence of bilayer surface), and the non-membranous morphology seen by TEM. This structural organization distinguishes NVEPs from canonical EVs and suggests they may function as specialized carriers for hydrophobic metabolic signals, distinct from the aqueous-cargo transport mediated by exosomes. To elucidate the molecular determinants of NVEP biogenesis, we interrogated the subcellular localization of EGFP-tagged markers in HEK293 cells by confocal microscopy coupled with an in-house developed membrane association assay. To dissect the biogenetic origins of distinct particle subpopulations, we selected a panel of markers representing defined intracellular trafficking routes: Arf6 (plasma membrane shedding/ectocytosis) [61], Rab7a (late endosomal/MVB sorting) [62], LC3b (secretory autophagy/amphisomes) [63], CD63 (canonical exosome biogenesis) [64], and a MysP motif to track direct plasma membrane budding [15]. This pathway-specific approach allowed us to correlate the intracellular localization of cargo with its sorting into distinct extracellular fractions.

Our analyses revealed that Arf6 and Rab7a exhibited perinuclear localization distinct from canonical endolysosomal markers CD63 and LC3b, as compared to a membrane-targeted control. Super-resolution dSTORM imaging confirmed the presence of classical tetraspanin markers—CD63, CD9, and CD81—on all extracellular vesicle (EV) subtypes. However, these markers were significantly less abundant on 167k-NVEPs, and lack CD81 suggesting a non-vesicular molecular composition. Similarly, Annexin V binding revealed negligible phosphatidylserine exposure in 167k-NVEPs, consistent with the lack of a typical bilayer membrane and the asymmetrical phospholipid distribution normally seen in EVs. Accordingly, Hsp90β was selected in this study as a marker for 167k-NVEPs, based on recent evidence demonstrating its relative enrichment in exomere-like particles [8, 65, 66]. We observed that no single marker—including Arf6, CD63, LC3b, MysP, and Rab7a—was uniquely restricted to a specific EV subtype or NVEPs, indicating that there is no marker exclusivity for the subtype but rather a heterogeneously distributed marker landscape. These marker profiles of the differently sized particle populations suggest contributions from distinct biogenetic routes: ectosomal shedding for larger 14k-lEVs, an MVB-associated pathway for 100k-sEVs, and late endosomal, secretory autophagy, or secretory lysosome–related mechanisms for the smaller sEVs and NVEPs. Together, these data indicate that particle size reflects the integration of multiple intracellular trafficking pathways rather than a single exclusive origin. Finally, dose-dependent uptake assays demonstrated that CD63-EGFP enriched NVEPs loaded at 500 µg total protein delivered EGFP to HEK293 recipient cells, underscoring NVEPs’ potential as non-membranous therapeutic vehicles. CD63-associated particles showed higher transfer efficiency, which may reflect the known role of CD63 in endosomal trafficking and cargo sorting. Consistent with this, soluble EGFP controls did not produce detectable signal transfer, supporting particle-mediated delivery rather than uptake of free protein. Together, detergent sensitivity analysis and immuno-EM findings suggest that CD63 detected in the 167k-NVEP may exist as protein-associated components of NVEPs rather than arising from membrane-bound EV contaminating the sample.

In summary, this study delineates 167k-NVEPs as a biochemically and structurally distinct class of extracellular nanoparticles, fundamentally separating them from canonical vesicular subtypes. Through orthogonal high-resolution profiling, we demonstrate that NVEPs are defined by a non-membranous lacking, lipoprotein-like architecture characterized by Annexin V signal, a high protein-to-lipid ratio, a dense protein shell enriched in adhesion markers (CD49e, CD56), and a hydrophobic core replete with metabolic storage lipids (CE, TG) and de novo synthesized fatty acids (C16/C18). This unique composition, coupled with the enrichment of Arf6 and the exclusion of classical tetraspanins (CD81), points to a non-canonical biogenetic origin at the nexus of secretory lipophagy and Arf6-mediated cortical extrusion, distinct from the ESCRT-dependent MVB pathway. By establishing a robust, label-free workflow to isolate and characterize these particles, we provide a framework for decoding the “non-vesicular” secretome, suggesting that NVEPs function not merely as byproducts, but as specialized metabolic and signaling modules with potential roles in long-range intercellular communication and tissue homeostasis, suggesting a potential translational application as a liquid biopsy biomarker for metabolic disorders. Future studies should investigate whether NVEP-associated cholesteryl esters are upregulated in patients with hypercholesterolemia or non-alcoholic fatty liver disease, offering a novel, non-lipoprotein readout for cellular cholesterol homeostasis and metabolic stress.

## Supplementary Information

Supplementary Material 1.

## Data Availability

All data needed to evaluate the conclusions in the paper are present in the paper and/or the Supplementary Materials and will be provided upon reasonable request.
