# Supplementary material for: Cholesteryl esters and high protein-to-lipid ratios distinguish non-vesicular extracellular particles from extracellular vesicles
Source: Cell Commun Signal. 2026 Apr 17;24:434. doi: 10.1186/s12964-026-02880-6 (PMC13450444; doi:10.1186/s12964-026-02880-6)
Supplement: Supplementary file 1 [file 12964_2026_2880_MOESM1_ESM.docx]

**Additional information for**

**Cholesteryl esters and high protein-to-lipid ratios distinguish Non-Vesicular Extracellular Particles from Extracellular Vesicles**

Sayam Ghosal *et al.*

*Corresponding author email: xabieruf@gmail.com

**This PDF file includes:**

Figures S1 to S14

Tables T1 to T4

Citations

**Fig. S1**

.

**Fig. S1. Schematic overview of the molecular cloning strategy**

(A) Assembly cloning was initiated using a construct obtained from Addgene to generate the pcDNA3_BRET vector, which served as the backbone for subsequent cloning steps. This vector was then used to generate constructs SGB42 (EGFP_ARF6) for downstream experiments, (B) SDM was further used to introduce a myristoylation and palmitoylation (MysP) cassette upstream of the EGFP coding region named as BMKb9 (MysP_EGFP). Details of the primers used for each cloning and mutagenesis step, as well as the final constructs, are provided in the Extended Table T1 and T2.

Fig. S2

Fig. S2 Image Processing and Analysis Pipeline

The entire pipeline (designed in draw.io) is shown, starting from the acquisition of fluorescence and brightfield images of individual 293A cells expressing various markers, such as Arf6, CD63, LC3b, MysP, and Rab7a. The pipeline was followed to perform the membrane association % analysis. The python script was run in Visual Studio Code Version: 1.106.2 (Universal). Images were pre-processed using Python, and contours were drawn from the nucleus and membrane. These contour images were analysed in CellProfiler 4.2.8, where the EGFP signal was segmented to determine the nucleus location and marker location (EGFP signal location in the cell). Distances between the nucleus and markers were calculated, generating a distance table using Python, and a membrane association plot was created using GraphPad Prism 10.3.0.

**Fig. S3**

**Fig. S3 Ultrastructural Characterization of morphology and dimension of EV subtypes and non-vesicular extracellular particles (NVEPs).**

Pellet based transmission electron microscopy (TEM) images of resin-sectioned samples derived from HEK293 cell serum-depleted conditioned media. (A) 14k-IEV complete field of view (FOV)-left, magnified - right, (B) 100k-sEV FOV-left, magnified - right, and (C) 167k-NVEP FOV-left, magnified - right fractions derived from HEK293 cells serum depleted media. 14k-IEV and 100k-sEV fractions exhibit clear lipid bilayer (red arrow) enclosed structure, while 167k-NVEP predominantly contains non-vesicular particles (green arrows). Scale bars: 100 nm (left panels), 50 nm (right panels).

**Fig. S4**

**Fig. S4 Characterization of individual EV subpopulations using multiplex bead-based flow cytometry and SP-IRIS.**

(A) Representative NTA size distribution profiles of PBS, 14k-lEV, 100k-sEV, and 167k-NVEP fractions acquired using instrument settings optimized for lEV and sEV measurements. While robust size distributions were obtained for 14k-lEVs and 100k-sEVs, the 167k-NVEP profile overlapped with PBS background and did not yield quantitatively interpretable data under these conditions. (B) Zeta potential measurements showing -28.07 ± 0.76 mV for 14k-IEV and -18.13 ± 10.20 mV for 100k-sEV (n=3 biological replicates). (C) Scatterplot depicting zeta potential as a function of particle size for individual 14k-IEV (cyan) and 100k-sEV (red) fractions (n=4 biological replicates). (D) Surface marker profiling of EV subpopulations—14k-lEV, 100k-sEV, and 167k-NVEP—was performed using multiplex bead-based flow cytometry (MACSPlex EV Human Kit). A representative gating strategy is shown, with PE-A vs. APC-A plots. Phosphate-buffered saline (PBS) served as a negative control, and gating thresholds were set to exclude background signals arising from the PBS region. (E) To enable inter-marker comparison, median APC-A fluorescence intensities were normalized by calculating the ratio of each marker signal to the average of canonical EV tetraspanins (CD9, CD63, and CD81). Following normalization, no substantial expression differences were observed for most markers across EV subtypes, except for CD146, which was elevated in 14k-lEV, and HLA-ABC, which was enriched in both 14k-lEV and 100k-sEV. (F) Among the 33 markers assessed, low-intensity APC-A signals were generally observed. Notably, CD146 expression was significantly higher in 14k-lEV, and HLA-DRDPDQ was enriched in 100k-sEV (p < 0.01, two-way ANOVA; n=3 biological replicates). (G) Single-particle interferometric reflectance imaging sensing (SP-IRIS) validated the presence of CD9, CD63, and CD81 on both 100k-sEV and 167k-NVEP subpopulations. No detectable signal was observed in the mIgG1 isotype control. A representative image from three independent biological replicates is shown. Data are presented as mean ± standard deviation, where applicable.

**Fig. S5**

**Fig. S5 dSTORM analysis of the EPs**

(A) Bar plot showing that native 167k-NVEPs has significantly less Annexin-V signal than 100k-sEV (p<0.0024, Two-tailed Unpaired t test, n=16). (B) Representative dSTORM images of 100k-sEV expressing CD63, where AnnexinV-positive and EGFP-positive signals are detectable. Each cluster was analyzed using the built-in EV profiling application of the CODI quantification software, which identifies subpopulations of single EV particles through a customized clustering workflow. This workflow first identifies clusters merging the signals of AnnexinV and EGFP. Here, a single 100k-sEV expressing CD63 contains 2964 AnnexinV-positive signals and 1580 EGFP-positive signals. Scale bar 5 mm (left) 200 nm (center and right).

**Fig. S6**

**Fig. S6 Single-particle interferometric reflectance imaging sensing (SP-IRIS) analysis of of lipoprotein contribution in the non-vesicular extracellular particles (NVEPs)**

(A)Size distribution and particle abundance measured by single-particle fluorescence analysis of 167k-NVEP preparations. Tetraspanin-positive particles (CD9/CD63/CD81; green) were detected across the nanoparticle size range, whereas ApoB-positive particles (pink), indicative of lipoprotein-associated particles, were detected at markedly lower abundance. The dashed line indicates 70 nm, corresponding to the diameter range in which most tetraspanin-positive 167k-NVEP particles were detected. (B) Diameter distribution of marker-positive particles. Tetraspanin-positive populations (CD9, CD63, CD81) exhibited significantly smaller diameters compared with ApoB-positive particles. Statistical significance was determined using ordinary one-way ANOVA with multiple comparisons (p < 0.0001). (C) Quantification of particle concentration showing tetraspanin-positive particles alone and in combination with ApoB labeling. Tetraspanin-positive particles were detected at several orders-of-magnitude higher abundance than ApoB-positive particles. Co-positive events represented a minor fraction of the detected population (p values indicated; ordinary one-way ANOVA). (D) Proportion of single-positive and double-positive particles within the analyzed population. Double-positive (tetraspanin+ApoB) events accounted for ~0.15% of total detected particles, indicating minimal contribution of ApoB-containing lipoproteins to the NVEP fraction.

Fig. S7

Fig. S7 Raman spectroscopic profiling of HEK293-derived extracellular vesicles.

(A) Representative Raman vibrational band assignments for HEK293-derived 100k-sEV and 167k-NVEP were based on established literature (1, 2). Spectral peaks correspond to characteristic molecular components such as lipids, nucleic acids, and proteins. (B) Comparative analysis of spectral fingerprints revealed significant differences between 100k-sEV and 167k-NVEP at several key Raman shifts: 708 cm⁻¹ (cholesterol ring deformation), 783 cm⁻¹ (nucleic acids), 1055 cm⁻¹ (Lipids I), 1130 cm⁻¹ (phenylalanine, protein side chain), 1210 cm⁻¹ (triacylglycerol), 1340 cm⁻¹ (glycosaminoglycans), 1441 cm⁻¹ (saturated lipids), 1556 cm⁻¹ (tryptophan), and 1720 cm⁻¹ (lipids), with p<0.05 for all comparisons (unpaired t-test; n=3 biological replicates).

Fig. S8

**Fig. S8** **Comprehensive lipidomic profiling of cells, 100k-sEV, and 167k-NVEP**

(A) Three-dimensional principal component analysis (PCA) plot illustrating distinct lipid signatures of cells (yellow), 100k-sEV (red), and 167k-NVEP (green). Each point represents an independent biological replicate (n=3). Axes correspond to principal components 1 (94.2%), 2 (4.9%), and 3 (0.6%), capturing 99.7% of total variance. Analysis includes eight EV-associated lipid classes: free cholesterol (FC), sphingomyelin (SM), phosphatidylethanolamine plasmalogen (PE-P), cholesteryl ester (CE), phosphatidylcholine (PC), phosphatidylethanolamine (PE), phosphatidylinositol (PI), and triglycerides (TG). Data were processed using MetaboAnalyst 5.0 with log transformation and Pareto scaling (FDR<0.05). Clustering aligns with MISEV2023 (3) guidelines for distinguishing EV subtypes and non-vesicular particles. (B) Relative abundance of specific lipid species across cells, 100k-sEV, and 167k-NVEP. Lipid species including phosphatidylcholine-ether (PC-O), phosphatidic acid (PA), lysophosphatidylethanolamine (LPE), lysophosphatidylcholine (LPC), hexosylceramide (HexCer), ceramide (Cer), phosphatidylserine (PS), phosphatidylglycerol (PG), diglyceride (DG), and cardiolipin (CL) show lower abundance in cells compared to their secreted 100k-sEV and 167k-NVEP counterparts. Values are normalized peak intensities (log2-transformed) from n=3 biological replicates.

**Fig. S9**

**Fig. S9. Characterization of Marker-Expressing EVs**

(A) LC3b, and Rab7a expressing 14k-lEVs showed significantly lower concentrations compared to their non-transfected counterparts (p=0.0057, 0.0098, and 0.0015, respectively, Ordinary one-way ANOVA). (B) All marker-expressing 100k-sEVs exhibited significantly lower concentrations compared to non-transfected 100k-sEVs (p<0.0001, Ordinary one-way ANOVA). (C) The mean size of all 14k-lEVs was 187.0 ± 34.99 nm. (D) All 100k-sEVs averaged 132.2 ± 13.50 nm, with no significant differences in sizes between any of the marker-expressing variants. (E) Total lipid of 14k-lEV per million Hek293 cells was significantly higher compared to both 100k-sEV and 167k-NVEP (p<0.0001, Two-way ANOVA, n=4). (F) Total protein of 167k-NVEP per million Hek293 cells was significantly higher compared to both 14k-lEV and 100k-sEV (p<0.05, Two-way ANOVA, n=4). (G) 167k-NVEPs showed have significantly high protein to lipid ratio than EV subtypes (p<0.01, Two-way ANOVA, n=4). Data are normalized to particles per mL per million cells to account for differences in cell number between transfected and non-transfected conditions.

Fig. S10

Fig. S10 High resolution Flow Cytometry Analysis of EVs

(A) Flow cytometry data were obtained from the Apogee A60 MP (SN 0130, Apogee Flow Systems, Northwood, UK) and analyzed using FlowJo 10.9.0 software. Gating was performed on native 14k-lEV and 100k-sEV samples that did not express any markers or EGFP. Signals from non-transfected 14k-lEV and 100k-sEV were excluded from the gating analysis, and a single representative plot is shown. Data represent n = 3 biological repeats. (B) Representative of high-resolution flow cytometry histograms showing single-vesicle detection of EGFP-tagged markers, including Arf6, CD63, LC3b, MysP, and Rab7a for 14k-IEV (cyan) and 100k-sEV (red) populations. (C) Percentage of EGFP-positive events for each marker in 14k-IEV and 100k-sEV, where similar distribution of data was observed. (D) Absolute counts of EGFP-positive events per mL for each marker, highlighting subtype-specific enrichment: LC3b, MysP in 14k-IEV and Rab7a, in 100k-sEV (unpaired two-tailed t test, p<0.01). Matrix dot plots showing mean of events/mL for each marker: Arf6 (black), CD63 (chartreuse), LC3b (green), MysP (purple), and Rab7a (coral) in 14k-IEV (d) and 100k-sEV (E). No marker was exclusive to a single subtype, but CD63 (48.43%) and MysP (38.16%) were more abundant in 14k-IEV, (F) while CD63 (79.28%) and Rab7a (13.47%) were more abundant in 100k-sEV. All data are shown as mean ± standard deviation from three independent experiments.

**Fig. S11**

**Fig. S11 Super-resolution marker profiling EVs and 167k-NVEPs reveal no exclusivity.**

Parts of whole matrix plots display the mean number of particles per field positive for each subcellular marker in 14k-lEVs (A), 100k-sEVs (B), and 167k-NEVPs (C), where the markers include Arf6 (black), CD63 (chartreuse), LC3b (green), MysP (purple), and Rab7a (coral). Notably, CD63 (29.77%), Arf6 (24.81%), MysP (25.57%) were enriched in 167k-NEVPs; Rab7a (37.15%) and CD63 (35.38%) in 100k-sEVs; and CD63 (61.89%), Rab7a (18.74%) and MysP (18.08%) in 14k-lEVs. While several markers showed subtype-specific enrichment, none were exclusive to a particular EV class. Rather, it is the relative enrichment pattern that distinguishes each vesicle population.

Fig. S12

**Fig. S12. Size Comparison of EVs Using NTA and dSTORM**

(A) Diameter of 100k-sEVs expressing different markers was compared using two orthogonal methods: NTA and dSTORM. The median diameter of 14k-lEVs expressing CD63 (190.70 ± 18.10 nm), MysP (215.80 ± 29.43 nm), and Rab7a (170.50 ± 4.81 nm) obtained by NTA was significantly higher than that measured by dSTORM (p<0.05, Mann-Whitney test). (B) A similar trend was observed for the median diameter measured by NTA for 100k-sEVs expressing CD63 (145.20 ± 8.29 nm) and MysP (158.10 ± 14.96 nm), which were significantly higher than the measurements obtained by dSTORM (p<0.05, Mann-Whitney test).

**Fig. S13**

**Fig. S13 Functional analysis of the 167k-NVEP reveals CD63 expressing 167k-NVEP’s potential to deliver CD63-EGFP to cells.**

(A) Schematic of NVEP uptake assay. HEK293 cells were incubated with increasing doses (1, 5, 20, 100, and 500 μg total protein) of 167k-NVEPs expressing different EGFP-tagged protein markers (Arf6, CD63, LC3b, MysP, Rab7a) and soluble EGFP as control. Following a 6-h incubation period, cellular uptake was assessed by quantifying EGFP-positive cells using flow cytometry. (B) Dose-dependent uptake efficiency of 167k-NVEPs according to marker identity. The percentage of EGFP-positive cells increased with increasing NVEP protein input for all marker-associated vesicles, whereas soluble EGFP showed minimal signal. Among the tested constructs, CD63-expressing 167k-NVEPs produced the highest level of cellular delivery, reaching 54.60 ± 0.95% EGFP-positive cells at 500 μg. Data are presented as mean ± standard deviation from three independent flow cytometry experiments (n = 3).

**Fig. S14**

**Fig. S14 Super-resolution and immunogold TEM analyses confirm CD63-positive vesicular structures in 100k-sEVs and 167k-NVEPs.**

(A) Quantification of double-positive clusters detected by dSTORM imaging. Samples corresponding to 100k-sEVs (red) and 167k-NVEPs (green) were analyzed in the absence or presence of detergent lysis (0.5% Triton X-100). Double-positive events were defined as spatially co-localized CD63 and Hsp90b antibody signals. Detergent treatment significantly reduced detectable clusters, consistent with membrane disruption and loss of vesicular integrity. (B) Diameter distribution of double-positive clusters measured by dSTORM under the indicated conditions. Particle size analysis revealed vesicle-scale structures for both 100k-sEVs and 167k-NVEPs, with a marked reduction following detergent lysis. (C–D) Immunogold transmission electron microscopy (TEM) of vesicles labeled for CD63. CD63 localization is indicated by 6-nm gold particles (black dots). Representative images show CD63-positive vesicles in 100k-sEV preparations (red arrowheads) and in 167k-NVEP samples (green arrowheads), confirming the presence of CD63 on membrane-bound particles in both fractions. Scale bar is 100 nm.

**Supplementary Table T1. Overview of primers utilized for assembly cloning and SDM.**

| I. | Vector (5’-3’) | Insert (5’-3’) |
| --- | --- | --- |
| a | GCAGCGGCGGCAGCGGCGGCGTGAGCAAGGGCGAGGAG | GGAGGATCAGGAGGCAGTGGTGGCGGTAC |
| b | CCACTGCCTCCTGATCCTCCCGCCAGAATGCGTTCGCAC | GCCGCCGCTGCCGCCGCT |
| c | GGAGGATCAGGAGGCAGTG | ACAAGGATATCTCCGGAGGTGGGAAGGTGCTATCCAAAATC |
| d | ACCTCCGGAGATATCCTTG | CCACTGCCTCCTGATCCTCCCACACTTCCAGCACTCGAG |
| II. | Forward Primer | Reverse Primer |
| a | CAAGAGAAAGGACGTGAGCAAGGGCGAGGAGC | CTGTTGCAGCAGCCCATCTCGAGCGGCCGCCA |

**Supplementary Table T2. Description of plasmids used, including specific single amino acid sequences. Distinct regions are highlighted with various colors for clarity.**

| Plasmid ID | Name and amino acid sequence |
| --- | --- |
| SGb42 | Myc_EGFP_Arf6_HA |
|  | EQKLISEEDLGSGGSGGSGGVSKGEELFTGVVPILVELDGDVNGHKFSVSGEGEGDATYGKLTLKFICTTGKLPVPWPTLVTTLTYGVQCFSRYPDHMKQHDFFKSAMPEGYVQERTIFFKDDGNYKTRAEVKFEGDTLVNRIELKGIDFKEDGNILGHKLEYNYNSHNVYIMADKQKNGIKVNFKIRHNIEDGSVQLADHYQQNTPIGDGPVLLPDNHYLSTQSALSKDPNEKRDHMVLLEFVTAAGITLGMDELYKDISGGGKVLSKIFGNKEMRILMLGLDAAGKTTILYKLKLGQSVTTIPTVGFNVETVTYKNVKFNVWDVGGQDKIRPLWRHYYTGTQGLIFVVDCADRDRIDEARQELHRIINDREMRDAIILIFANKQDLPDAMKPHEIQEKLGLTRIGSAGSVGGSGGSGGGTYPYDVPDYA |
| BMKb9 | MysP-EGFP |
|  | MGCCNSKRKDVSKGEELFTGVVPILVELDGDVNGHKFSVSGEGEGDATYGKLTLKFICTTGKLPVPWPTLVTTLTYGVQCFSRYPDHMKQHDFFKSAMPEGYVQERTIFFKDDGNYKTRAEVKFEGDTLVNRIELKGIDFKEDGNILGHKLEYNYNSHNVYIMADKQKNGIKVNFKIRHNIEDGSVQLADHYQQNTPIGDGPVLLPDNHYLSTQSALSKDPNEKRDHMVLLEFVTAAGITLGMDELYK |

Color denotation:

|  | EGFP |
| --- | --- |
|  | Markers |
|  | Myc affinity tag |
|  | HA affinity tag |

**Supplementary Table T3. Internal standards used for lipid class quantification**

| Lipid class | Internal standard(s) |
| --- | --- |
| Phosphatidylcholine (PC) | PC 28:0; PC 44:0 |
| Lysophosphatidylcholine (LPC) | LPC 13:0; LPC 19:0 |
| Phosphatidylethanolamine (PE) | PE 28:0; PE 40:0 |
| Lysophosphatidylethanolamine (LPE) | LPE 13:0; LPE 18:1[D7] |
| Phosphatidylserine (PS) | PS 33:1[D7]; PS 40:0 |
| Lysophosphatidylserine (LPS) | LPS 13:0 |
| Phosphatidylglycerol (PG) | PG 28:0; PG 40:0 |
| Phosphatidylinositol (PI) | PI 33:1[D7] |
| Lysophosphatidylinositol (LPI) | LPI 13:0 |
| Sphingomyelin (SM) | SM 30:1;O2; SM 18:1;O2/18:1[D9] |
| Ceramide (Cer) | Cer 14:0; Cer 18:1;O2[D7]/18:0 |
| Hexosylceramide (HexCer) | HexCer 30:1;O2; HexCer 18:1;O2[D5]/18:0 |
| Diacylglycerol (DG) | DG 28:0; DG 40:0 |
| Triacylglycerol (TG) | TG 51:0; TG 57:0 |
| Cholesteryl ester (CE) | CE 17:0; CE 22:0 |
| Free cholesterol (FC) | FC[D7] |
| Fatty acids (FA) | FA 13:0; FA 19:0 |
| Phosphatidic acid (PA) | PA 28:0 |
| Cardiolipin (CL) | CA (14:0)₄ |

**Supplementary Table T4. Lipid class composition (mol%) of cells, 100k-sEVs, and 167k-NVEPs**

| Lipid_Class | Cell1 | Cell2 | Cell3 | 100k-sEV1 | 100k-sEV2 | 100k-sEV3 | 167k-NVEP1 | 167k-NVEP2 | 167k-NVEP3 |
| --- | --- | --- | --- | --- | --- | --- | --- | --- | --- |
|  | mole percentage | | | | | | | | |
| PC O | 4.259 | 4.293 | 4.395 | 3.960 | 4.295 | 3.021 | 1.836 | 1.090 | 2.329 |
| PC | 24.420 | 23.631 | 24.736 | 8.406 | 8.727 | 9.171 | 12.033 | 11.404 | 12.706 |
| SM | 3.793 | 3.796 | 3.623 | 10.870 | 12.435 | 10.470 | 8.910 | 7.343 | 7.318 |
| DG | 3.182 | 2.935 | 2.744 | 0.935 | 0.805 | 0.840 | 0.246 | 0.106 | 0.192 |
| TG | 13.215 | 13.383 | 12.311 | 0.427 | 0.494 | 0.827 | 0.643 | 0.461 | 0.500 |
| CE | 7.882 | 5.671 | 6.159 | 6.455 | 8.087 | 3.353 | 30.634 | 51.963 | 42.036 |
| FC | 20.453 | 20.160 | 20.657 | 57.628 | 53.548 | 60.206 | 40.805 | 25.444 | 31.632 |
| LPC | 0.824 | 1.232 | 0.804 | 0.393 | 0.467 | 0.493 | 0.318 | 0.291 | 0.291 |
| LPE | 0.316 | 0.436 | 0.368 | 0.367 | 0.385 | 0.474 | 0.044 | 0.000 | 0.025 |
| Cer | 0.196 | 0.203 | 0.192 | 0.239 | 0.286 | 0.189 | 0.121 | 0.072 | 0.086 |
| PA | 0.203 | 0.211 | 0.235 | 0.119 | 0.166 | 0.195 | 0.011 | 0.006 | 0.007 |
| HexCer | 0.156 | 0.165 | 0.154 | 0.659 | 0.682 | 0.571 | 0.287 | 0.205 | 0.275 |
| PE | 7.942 | 8.581 | 8.382 | 0.657 | 0.552 | 1.257 | 0.028 | 0.008 | 0.008 |
| PS | 2.494 | 2.590 | 2.510 | 3.079 | 2.970 | 3.635 | 1.508 | 0.105 | 0.414 |
| PG | 0.101 | 0.103 | 0.100 | 0.014 | 0.018 | 0.010 | 0.005 | 0.004 | 0.005 |
| PE P | 2.208 | 2.303 | 2.259 | 4.409 | 4.498 | 4.105 | 2.205 | 1.254 | 1.859 |
| PI | 7.357 | 9.255 | 9.330 | 1.346 | 1.553 | 1.171 | 0.310 | 0.188 | 0.282 |
| CL | 0.998 | 1.052 | 1.042 | 0.037 | 0.032 | 0.012 | 0.055 | 0.057 | 0.034 |

**Reference:**

1.	Rygula A, Majzner K, Marzec KM, Kaczor A, Pilarczyk M, Baranska M. Raman spectroscopy of proteins: a review. Journal of Raman Spectroscopy. 2013;44(8):1061–76.

2.	Czamara K, Majzner K, Pacia MZ, Kochan K, Kaczor A, Baranska eM. Raman spectroscopy of lipids: a review. Journal of Raman spectroscopy. 2015;46(1):4–20.

3.	Welsh JA, Goberdhan DCI, O'Driscoll L, Buzas EI, Blenkiron C, Bussolati B, et al. Minimal information for studies of extracellular vesicles (MISEV2023): From basic to advanced approaches. J Extracell Vesicles. 2024;13(2):e12404.
